# On the Empirical Agreement Between Compression and Program-Execution Approaches to Algorithmic Complexity: A Controlled Study Using BDM

**DOI:** 10.3390/e28060601

**Published:** 2026-05-27

**Authors:** Zoe Leyva-Acosta, Eduardo Acuña Yeomans, Francisco Hernández-Quiroz

**Affiliations:** 1 Posgrado en Ciencia e Ingeniería de la Computación, Universidad Nacional Autónoma de México, Mexico City 04510, Mexico; eduardo.acuna@unison.mx; 2 Departamento de Matemáticas, Universidad de Sonora, Hermosillo 83000, Mexico; 3 Facultad de Ciencias, Universidad Nacional Autónoma de México, Mexico City 04510, Mexico; fhq@ciencias.unam.mx

**Keywords:** algorithmic complexity, Coding Theorem Method, Block Decomposition Method, compresion-based estimates

## Abstract

Algorithmic complexity is a foundational notion in theoretical computer science, but its incomputability has led to two families of practical estimators: compression-based and program-execution-based (e.g., the Coding Theorem Method, CTM). Despite widespread use, the correspondence between these paradigms remains poorly understood. We present a systematic comparative framework that uses the Block Decomposition Method (BDM) to extend CTM-based estimates to longer strings, enabling direct comparison with compression-based estimators across multiple computational models. A control estimator (BDM_Id_) isolates the contribution of block structure from algorithmic information, providing a rigorous baseline for interpreting correlations. Our results show that cross-paradigm correlations are weak and decrease systematically as model resolution decreases; for the lowest-resolution model, correlations are essentially null. In long strings, per-length correlations vanish, while global correlations appear high but are largely explained by the control estimator, indicating that they are driven primarily by trivial length effects rather than shared sensitivity to algorithmic structure. Crucially, for low-resolution models, BDM_Id_ outperforms BDM itself, indicating that the inclusion of CTM information does not improve—and may even reduce—agreement with compression-based estimators. These findings suggest that compression-based and program-execution-based estimators capture fundamentally different aspects of structure. Rather than invalidating either approach, this work provides a systematic methodology for assessing cross-paradigm correspondence and highlights the importance of explicit controls in empirical comparisons of algorithmic complexity.

## 1. Introduction

Algorithmic complexity, also known as Kolmogorov complexity, is a foundational notion in theoretical computer science and information theory. In its prefix-free formulation, the complexity of a finite binary string *x*, denoted K(x), is defined as the length of a shortest prefix-free program that outputs *x* when executed on a universal reference machine [1]. This definition provides a mathematically rigorous measure of the information content of individual objects without assuming any probability distribution.

Intuitively, strings exhibiting regularities admit shorter descriptions than those lacking exploitable structure. A highly regular string can be generated by a concise program, whereas a string devoid of regular patterns requires a program that essentially stores the entire string explicitly. In this sense, algorithmic complexity formalizes the idea that structure corresponds to compressibility in a generative sense.

Despite its conceptual elegance, K(x) faces a fundamental limitation: it is not computable. As a consequence, several practical approximation approaches have been suggested. These can be broadly classified into two paradigms. The first consists of compression-based estimators, which approximate complexity through the length of a compressed representation using general-purpose lossless compression algorithms [2,3,4,5]. The second comprises program-execution-based estimators, which approximate complexity via algorithmic probability by systematically exploring the space of programs and outputs for a fixed computational model, most notably through the Coding Theorem Method (CTM) and related techniques [6,7,8,9,10].

Although both paradigms are motivated by the theoretical framework of algorithmic information theory, they rely on fundamentally different operational principles. Compression algorithms are optimized to detect statistical redundancy and local repetition, whereas program-execution approaches aim to capture generative algorithmic structure through explicit enumeration of programs. Furthermore, while the invariance theorem ensures that the difference between the complexities under two reference machines is bounded by an additive constant asymptotically, such a guarantee does not necessarily eliminate meaningful discrepancies in the finite domains where practical approximations are computed.

These approaches have been widely applied in empirical contexts; however, comparatively little attention has been devoted to systematically evaluating their mutual correspondence. Existing comparative studies suggest partial agreement, reporting that strings of higher complexity as assigned by CTM tend to be less compressible [11,12]. However, these analyses typically rely on restricted experimental settings, limited model diversity, and methodological choices that may introduce significant artefacts. A notable example is the practice of concatenating strings of known CTM complexity to construct files long enough for effective compression, and then evaluating the compressibility of these constructed artefacts. Such strategies, while pragmatically motivated by the computational infeasibility of CTM for long strings and the ineffective performance of compression algorithms for very short ones, may obscure or distort the relationship between the estimators themselves. Systematic and symmetric evaluation of agreement across different estimation paradigms, explicit control for trivial length effects, and comparison across distinct computational models remain comparatively underexplored. Recent surveys of complexity estimation methods [13] explicitly identify the systematic comparison across paradigms—and the lack of agreed-upon validation strategies—as an open problem in the field. The present study addresses this gap by providing a controlled, multi-model, symmetrical empirical comparison that introduces a structure-only baseline (BDM_Id_), allowing us to distinguish trivial length- and block-effects from genuine algorithmic agreement.

The central question addressed in this work is, therefore, the following: How do compression-based and program-execution-based estimators compare as practical approximations of algorithmic complexity? Do they capture the same structural features, or do they reflect fundamentally different aspects of string organization?

To answer this question, we develop a unified comparative framework designed to minimize methodological asymmetries between paradigms. Our approach incorporates multiple representative estimators from both families, extensions of CTM via the Block Decomposition Method (BDM), carefully configured compression algorithms, and multiple reference computational models. Correlation is evaluated using rank-based statistical measures, with particular attention to the positionally unambiguous regime where BDM estimates are free from the confounding effects of non-consecutive repetitions. A control estimator (BDM_Id_) is introduced to isolate the contribution of block structure from algorithmic information, providing a rigorous baseline against which to interpret cross-paradigm correlations. Experiments are conducted on both exhaustive finite domains and on randomized samples of longer strings.

By providing a systematic and statistically grounded assessment, this study characterizes the relationship between these two families of estimators, showing that they capture largely different aspects of string structure. The weak correspondence observed is largely attributable to trivial features—string length and block structure—while the contribution of algorithmic information remains modest and model-dependent. These findings do not invalidate either approach; rather, they clarify their respective contexts of applicability and underscore the need for careful controls when interpreting cross-paradigm comparisons.

## 2. Background and Theoretical Framework

### 2.1. Plain Algorithmic Complexity vs. Prefix Complexity

The plain algorithmic complexity (or Kolmogorov–Chaitin complexity) of a string *s*, denoted by $C_{U}\left(s\right)$, is defined as the length of the shortest program *p* that, when executed on a universal Turing machine *U*, produces *s* and halts. Formally:

(1) $$C_{U}\left(s\right)=\min\left\{|p|:U\left(p\right)=s\right\}$$

where |p| represents the length of *p* measured in bits.

Prefix complexity, denoted by $K_{U}\left(s\right)$, is a variant of Kolmogorov complexity that imposes an additional constraint: the set of valid programs must form a prefix-free set, meaning no program can be a prefix of another. This constraint ensures that programs are self-delimiting, allowing for the unambiguous concatenation of multiple program descriptions without separator markers. Formally, prefix complexity is defined as:

(2) $$K_{U}\left(s\right)=\min\left\{|p|:U\left(p\right)=s\;\mathrm{and}\;\mathrm{the}\;\mathrm{domain}\;\mathrm{of}\;U\;\mathrm{is}\;\mathrm{prefix}\text{-}\mathrm{free}\right\}$$

Throughout this work, prefix complexity *K* serves as the underlying theoretical reference, rather than plain complexity *C*. The two quantities differ by at most O(log|s|) for binary strings, but several practical and theoretical considerations make *K* the natural framework for the present study. First, self-delimitation makes *K* additive under concatenation up to a constant, which is essential when reasoning about decomposition-based estimators such as BDM, where local complexities of blocks are combined by addition. Second, CTM is designed to approximate K(s) by construction, via the Coding Theorem and the algorithmic probability framework. Third, lossless compressors often produce prefix-free codes, so the compressed length they output is naturally interpreted as an upper bound on *K* rather than on *C*.

#### Invariance Theorem and Its Implications

The Invariance Theorem establishes that algorithmic complexity is robust across different choices of universal Turing machines. Formally, for any two universal Turing machines *U* and *V*:

(3) $$|C_{U}\left(s\right)-C_{V}\left(s\right)|\leq c_{U,V}$$

where $c_{U,V}$ is a constant that depends on *U* and *V* but is independent of the string *s*.

The invariance theorem has profound implications. First, it establishes algorithmic complexity as an intrinsic property of the object being measured, rather than an artifact of the specific computational model used for measurement. This objectivity creates a foundation for comparing the complexity of different objects independently of computational framework.

Second, the theorem legitimizes the practice of fixing a particular universal Turing machine as a reference and defining complexity relative to this machine, with the assurance that a different choice of reference machine would alter complexity values by at most a constant. This convention allows us to write C(s) instead of $C_{U}\left(s\right)$ when the specific choice of universal machine is not critical to the theoretical aspects of our discussion.

Third, the invariance theorem guarantees the asymptotic convergence of complexity values across different reference machines. As string length increases, the constant difference between complexity values becomes increasingly negligible relative to the complexity itself.

However, the theorem has important limitations in practical applications. While it ensures asymptotic invariance, it provides no guarantees about convergence rates. The additive constant may be arbitrarily large, and in practical implementations, understanding the sensitivity of complexity estimations to changes in the reference machine becomes crucial. These considerations are particularly relevant when developing approximation methods for Kolmogorov complexity.

### 2.2. Algorithmic Complexity Approximations

Kolmogorov complexity is a theoretical construct that is not computable. Consequently, no practical approximation method can claim to produce a “ground truth” value to which other estimators should aspire. This fundamental limitation has important implications for empirical work. Furthermore, algorithmic complexity is inherently defined only up to an additive constant. This means that the absolute numerical value of an approximation is less meaningful in practice than the relative ordering it imposes on strings. This observation motivates the use of rank-based correlation measures in comparative studies, as they focus on the consistency of orderings rather than absolute numerical agreement.

There are currently two main approaches for estimating algorithmic complexity. The program-execution-based approach relies on the systematic execution of a set of programs from a specific reference machine in the search for a shortest program producing some strings, or an estimation of the length of such a program. The compression-based approach relies on the use of a lossless compressor that attempts to exploit statistical regularities in strings to produce even shorter strings. When using compressors, the length of the compressed string is used as the approximation, but with the execution of programs, some methods rely on the output frequency distribution of strings to yield fractional approximations. In order to accommodate both approaches, we define a complexity estimator $\bar{K}$ as a partial function that assigns non-negative real values to binary strings:

(4) $$\bar{K}:{\left\{0,1\right\}}^{*}\to R^{+}$$

### 2.3. Program-Execution-Based Methods

The program-execution based approach performs expensive computations to produce a table of approximated complexities for a finite set of strings, which is later used as an efficient lookup table for applications. Compared to its counterpart, this approach is considerably more limited in that the complexity estimations it is currently able to provide are only for strings up to 12 or 13 bits long.

#### 2.3.1. The Coding Theorem Method (CTM)

The Coding Theorem Method (CTM) [6,7,8,14] is a numerical approach designed for approximating the algorithmic complexity of short strings. It relies on the relationship between algorithmic probability and prefix-free Kolmogorov complexity, formally established by the algorithmic Coding Theorem [1]. The method consists of simulating all Turing machines within a finite space, typically defined by the number of states *n* and symbols *m*, denoted as the (n,m) space. Each Turing machine $T_{p}$ in this space is run on an initially blank tape, and its output is recorded if the machine halts. The frequency of production of each string *x* as an output among all halting machines is interpreted as an empirical approximation of its algorithmic probability. Then, let |(n,m)| denote the total number of Turing machines in the space (n,m), the relative frequency of production $D_{\left(n,m\right)}\left(x\right)$ of a string *x* is defined as:

(5) $$D_{\left(n,m\right)}\left(x\right)=\frac{\left|\{p\in \left[1,|\left(n,m\right)|\right]:T_{p}\left(\varepsilon \right)=x\}\right|}{\left|\{p\in \left[1,|\left(n,m\right)|\right]:T_{p}\;\mathrm{halts}\}\right|}$$

where $T_{p}\left(\varepsilon \right)$ denotes the output of the *p*-th Turing machine on empty input.

Then, the algorithmic prefix-free complexity of a string *x* is approximated by the negative logarithm of its algorithmic probability:

(6) $${\mathrm{CTM}}_{\left(n,m\right)}\left(x\right)=-\log_{2}D_{\left(n,m\right)}\left(x\right)$$

#### 2.3.2. The Block Decomposition Method (BDM)

To extend CTM-based approximations beyond very short strings, the Block Decomposition Method (BDM) was introduced by Zenil et al. [15]. BDM decomposes a longer string into smaller blocks for which CTM values are available and combines these local estimates additively, incorporating a logarithmic term to account for block multiplicities.

Formally, given a decomposition of a string into blocks $\left\{b_{i}\right\}$ of size *l*, the BDM estimate is defined as:

(7) $$BDM_{M(l)}\left(s\right)=\sum_{i}\left[CTM_{M}\left(b_{i}\right)+\log_{2}\left(n_{i}\right)\right],$$

where $n_{i}$ denotes the multiplicity of block $b_{i}$ in the decomposition. In this way, BDM blends local algorithmic estimates with a global accounting of repetitions.

BDM is a hybrid technique that inherits the generative perspective of CTM but introduces structural assumptions through the chosen block size and decomposition strategy. These parameters influence the balance between local algorithmic structure and global repetition and, therefore, affect the resulting estimates in finite domains.

### 2.4. Compression-Based Complexity Estimators

Lossless compression algorithms provide practical tools for approximating Kolmogorov complexity, based on the theoretical connection between compressibility and algorithmic complexity. If $\hat{C}\left(x\right)$ represents the length of string *x* when compressed by algorithm $\hat{C}$, then $\hat{C}\left(x\right)$ serves as an upper bound for the Kolmogorov complexity K(x), up to a constant that depends on the compression algorithm. This relationship follows from the observation that a decompressor paired with a compressed representation constitutes a program that reproduces the original string.

Lossless compressors are designed to exploit statistical regularities in data, primarily repetitions and frequency distributions. This design focus means they excel at detecting statistical patterns but miss algorithmic patterns that do not manifest as statistical regularities. As a result, compression algorithms function more as estimators of Shannon entropy than true algorithmic complexity.

Additionally, compression algorithms struggle with short strings. When compressing short data sequences, the overhead of encoding the decompression instructions often exceeds any potential savings from compression. This limitation is particularly relevant when studying small binary strings, where the theoretical differences between entropy and algorithmic complexity are most pronounced.

Despite these limitations, compression-based approximations have been widely adopted in practice due to their computational efficiency, availability, and reasonable performance on data with predominantly statistical regularities.

## 3. Analytical Framework and Experimental Methodology

The two main approaches to approximating algorithmic complexity embody fundamentally different trade-offs. Compression-based methods are computationally efficient and scalable, but their design—optimized for detecting statistical redundancy—may cause them to miss algorithmic patterns that do not manifest as frequency-based regularities. Program-execution-based methods, by contrast, have the potential to uncover more complex algorithmic structure through systematic exploration of program space. However, they face significant computational challenges: the vast space of possible programs and the non-halting problem limit their applicability to very short strings (typically up to 12 bits).

### 3.1. The Validation Problem in Algorithmic Complexity Approximation

Validating any approximation to algorithmic complexity confronts a fundamental difficulty: due to the incomputability of K(x) and its asymptotic nature, derived from the unavoidable dependence on a reference computational model [16], there exists no “ground truth” value against which estimates can be compared. In the absence of such a reference, the literature has adopted indirect validation strategies that vary considerably across approaches.

In most works proposing compression-based methods, validation proceeds pragmatically: complexity estimates are used to construct similarity or distance measures, which are then evaluated on concrete applications—typically clustering problems in diverse domains [2,3,4,5]. In these studies, the criterion for validity is the successful performance of the resulting metric on the task at hand, compared to standard alternative methods in the application domain. For program-execution-based methods, by contrast, validation has tended to focus on the stability of estimates across different reference machines, using Spearman’s rank correlation coefficient as the primary tool [6,11,14,17]. Program-execution-based estimators, particularly those based on CTM and BDM, have also been applied successfully across diverse domains including biological sequences [18], pattern classification [19], and network analysis [11,20].

### 3.2. Methodological Limitations of Previous Comparative Studies

Studies that directly address the correspondence between compression-based and program-execution-based estimates are scarce, but important contributions exist [11,12,13]. These works report evidence of consistency between approaches: strings with higher complexity according to CTM tend to be less compressible than those with lower CTM values, a pattern that holds across different compression algorithms.

Despite encouraging evidence of partial correspondence, several methodological aspects remain comparatively underexplored. First, while rank correlation has been used to assess stability within the program-execution paradigm, no quantitative correlation analysis has been applied to the comparison between compression-based and program-execution-based estimates. This omission is significant because rank correlation offers a principled way to assess agreement in relative orderings—precisely the aspect of complexity that is theoretically meaningful given the additive constant freedom of K(x). Second, the analysis presented is not fully symmetric. The emphasis is on demonstrating that strings with low CTM complexity are more compressible, but the converse direction—whether more compressible strings systematically correspond to lower CTM values—is not examined with equal systematicity. This asymmetry limits the scope of the conclusions. Third, most studies rely on a single reference computational model, leaving open the question of whether observed agreement is robust under model variation in finite domains. Fourth, these studies do not discuss in detail crucial technical aspects: the conversion from bit strings to byte strings required for applying compressors and other configurations. Since compression algorithms are designed to operate on byte-oriented data, the bit-packing strategy (padding, streaming, ASCII representation) can affect not only absolute compressibility values but, more importantly, the sensitivity and variability of the estimates, directly influencing the feasibility and results of comparative analyses.

In summary, the existing literature provides a valuable first approximation to comparing complexity estimation paradigms, but leaves several questions open: (i) the absence of a direct quantitative correspondence analysis using tools such as Spearman’s correlation; (ii) the asymmetry of previous analyses, which do not explore both directions of the relationship with equal depth; (iii) the restriction to a single reference computational model; and (iv) the lack of systematic investigation into how compressor configuration and input representation affect estimate sensitivity.

The present work addresses these limitations through a multi-model, rank-based, systematic comparative methodology with explicit control mechanisms designed to distinguish structural agreement from trivial correlational effects. Additionally, we explicitly assess compressor configuration and sensitivity in the short-string domain to determine the applicability of rank-based analysis; and employ BDM as a bridging estimator to extend comparisons to longer strings without resorting to concatenation artifacts. These conceptual differences between our approach and previous comparative studies account for the empirical discrepancies we report, such as the weak correlations, which might otherwise appear as contradictions.

### 3.3. BDM as a Bridging Strategy

As it has been noted before [11,12], a fundamental challenge in comparing program-execution-based methods with compression-based estimators is the disparity in their operational domains. The former provide direct complexity estimates for strings up to 12 bits, a regime where compression algorithms are known to be ineffective due to header overhead and lack of significant statistical redundancy. The authors in previous studies addressed this challenge by attempting to “bring compressors down” to the short-string domain: They construct different files by concatenating 100 strings of equal length and known CTM complexity, but with a crucial encoding step: each string is represented using a distinct pair of symbols for ‘0’ and ‘1’, drawn from a common pool of 200 symbols. This encoding is intended to prevent compressors from exploiting regularities across different strings in the same file, isolating the compressibility of individual strings. This strategy, while pragmatically motivated and ingenious, transforms the object of measurement in ways that are difficult to fully characterize: boundary and alignment effects, alphabet expansion, and order dependencies. In essence, it asks compressors to operate indirectly where they are naturally weak, and then interprets the results as bearing on the relationship between the original estimators.

Furthermore, this strategy rests on an implicit assumption that compressors are necessarily insensitive in the short-string domain. However, the fact that a compressor fails to significantly reduce the size of a short string in absolute terms does not imply that it lacks the sensitivity to distinguish between strings of different complexity. If compressed lengths reflect, even to a small degree, the regularities present in the original strings, a less indirect strategy, one that avoids concatenation artifacts, might be feasible.

The present study adopts a fundamentally different strategy. Rather than forcing compressors into a domain where they are inherently weak, we instead extend the program-execution paradigm upward toward longer strings using the Block Decomposition Method (BDM). This method provides a transparent and principled way to estimate complexity for strings of moderate length, beyond the direct CTM regime. Through validation against CTM, it preserves the generative perspective of algorithmic complexity. This allows us to compare both families of estimators on identical strings of sufficient length for compressors to exhibit meaningful variability.

#### 3.3.1. Validating BDM Against CTM and Theoretical Expectations

Before BDM can serve as a reliable bridge between paradigms, its internal consistency must be established. Since CTM values are only available for strings up to a model-specific maximum length $b_{\max}$ (the maximum length covered exhaustively by the CTM table), we cannot directly validate BDM against CTM for longer strings. Instead, we assess the stability of BDM estimates under variations in block size using rank correlation. Stability in this context refers to the degree to which rankings induced by BDM remain consistent when the block decomposition parameter is varied.

For a given computational model *M* with CTM table covering all strings up to length $b_{\max}\left(M\right)$, we compute BDM estimates for strings of various lengths using different block sizes $b\leq b_{\max}\left(M\right)$. We then compare, via Spearman’s rank correlation, the estimates obtained with block size *b* against those obtained with the maximum block size $b_{\max}\left(M\right)$. High correlation indicates that the ordering induced by BDM is robust to the choice of block size, and that $b_{\max}\left(M\right)$ preserves the relative complexity structure captured by finer-grained decompositions.

This validation step serves two purposes. First, it provides empirical grounds for selecting $b_{\max}\left(M\right)$ as the block size for subsequent analyses with model *M*, since it maximizes the information available from CTM while maintaining consistency with other block sizes. Second, it quantifies the extent to which BDM’s ordinal rankings are stable under decomposition variation, which is essential for interpreting cross-paradigm correlations.

#### 3.3.2. String Length Regimes and BDM Fidelity

The faithfulness of BDM as an estimator of algorithmic complexity depends critically on the relationship between string length *L* and block size *b*, as well as on the structure of repetitions within the string. Zenil et al. [15] motivate the BDM formula by noting that for a string consisting of *s* consecutive repetitions of a block *x*, the algorithmic complexity can be approximated as K(x)+O(logs) bits since a description can specify *x* once and then indicate that it is repeated *s* times. This observation justifies the $\log_{2}\left(n_{i}\right)$ term in BDM for blocks that appear multiple times, but only when the repetitions are consecutive.

A crucial and often overlooked limitation arises when blocks repeat in non-consecutive positions. Consider a string of three complete blocks, $\left[b_{1}\right]\left[b_{2}\right]\left[b_{1}\right]$, where block $b_{1}$ appears at positions 1 and 3. BDM assigns this string a complexity of:

(8) $$BDM=CTM\left(b_{1}\right)+CTM\left(b_{2}\right)+\log_{2}\left(2\right),$$

treating the two occurrences of $b_{1}$ identically to how it would treat consecutive repetitions $\left[b_{1}\right]\left[b_{1}\right]$. However, this ignores the additional information required to specify that the second occurrence of $b_{1}$ is separated by $b_{2}$, rather than immediately following the first. In algorithmic information theory, this positional information has a cost that is not captured by the simple $\log_{2}\left(n_{i}\right)$ term.

This limitation motivates a careful distinction between string length regimes relative to the block size *b*:

- Direct validation regime (L≤b): Strings are shorter than or equal to the block size. BDM reduces to CTM, allowing direct validation against the reference method.
- Positionally unambiguous regime (b<L<3b): Strings are shorter than three complete blocks.

**Remark** **1.** *In this regime, any repetition of a complete block necessarily occurs in consecutive positions. Blocks of length less than b are treated as distinct types by BDM, so they cannot create non-consecutive repetitions with complete blocks. This regime, therefore, avoids the positional ambiguity that affects longer strings where three or more complete blocks can repeat in non-consecutive positions.*

- Positionally ambiguous regime (L≥3b): Strings decompose into three or more complete blocks. Here, non-consecutive repetitions between blocks of equal length become fully possible, and the positional information required to specify the arrangement of repeated blocks is not captured by BDM’s $\log_{2}\left(n_{i}\right)$ term.

Several consequences follow from this distinction and shape the interpretation of our results. First, in the positionally ambiguous regime BDM is expected to systematically underestimate the complexity of strings whose repeated blocks occur in non-consecutive positions, since two arrangements that differ only in the positions of the repetitions—say $\left[b_{1}\right]\left[b_{2}\right]\left[b_{1}\right]$ vs. $\left[b_{1}\right]\left[b_{1}\right]\left[b_{2}\right]$—are assigned the same BDM value despite carrying different amounts of positional information. Second, this bias is expected to grow with string length: as L/b increases, the number of distinct arrangements compatible with a given multiset of blocks grows combinatorially, while BDM’s value remains invariant under permutations of the blocks. Third, the bias affects BDM and compression-based estimators asymmetrically: standard compressors (e.g., LZ77, Deflate) are sensitive to the relative position of repeated patterns through their window-based parsing, so cross-paradigm agreement may decrease in the positionally ambiguous regime not only because of trivial length effects but also because the two paradigms encode positional information differently. We return to this point in the discussion of long-string results, and we flag the empirical characterization of BDM in the regime L≥3b for the highest-resolution model (TM, b=12, i.e., L≥36) as a direction that this study does not resolve and that we treat as a priority for future work.

In this study, we leverage the positionally unambiguous regime (L<3b) as a privileged domain for comparing BDM with compression-based estimators. This regime balances two desirable properties: (i) it extends beyond the direct CTM domain, allowing compressors to exhibit meaningful sensitivity; and (ii) it avoids the positional ambiguity that complicates interpretation in longer strings, enabling any observed agreement with compression to be interpreted more directly as evidence of shared sensitivity to algorithmic structure.

### 3.4. Estimator Sensitivity vs. Compression Rate

A common default criterion for selecting compression algorithms as proxies for algorithmic complexity is their compression rate. However, this criterion might be related to one of the most frequently cited shortcomings of compression-based estimators. In particular, it has been argued that these approaches perform poorly on short strings, since the compressed versions of such strings often end up longer than the originals [11,12]. This line of reasoning might suggest that achieving a higher compression rate necessarily implies a better-suited complexity estimator.

However, relying on compression rate as the sole criterion for algorithm selection overlooks a critical property of robust complexity estimators: their sensitivity. Sensitivity refers to the ability to meaningfully distinguish between strings of different complexity levels, regardless of the absolute values assigned to them. We argue that compression rate itself is not the primary concern, since even a compressor that produces outputs longer than its inputs can serve as a valid estimator, provided the resulting lengths reliably reflect the statistical regularities present in the data.

The sensitivity or variability of the estimator is a key aspect of our methodology, which deemphasizes the compression rate or encoding efficiency of the estimator. This approach aims to overcome the perceived shortcomings of compression-based estimators when estimating the complexity of short strings. This focus on sensitivity rather than absolute compression rate directly informs our application of rank-based correlation analysis.

#### Criteria for Estimator Sensitivity Under Incompressibility Constraints

A naive interpretation of estimator sensitivity would equate high variability with estimator quality. However, algorithmic information theory (AIT) imposes strong structural constraints on the distribution of complexity values over fixed-length domains. In particular, for every constant *c*, the number of *c*-incompressible strings of length *n* satisfies

(9) $$|\{x\in {\left\{0,1\right\}}^{n}:K\left(x\right)\geq n-c\}|\geq 2^{n}-2^{n-c}+1,$$

implying that the overwhelming majority of strings are incompressible up to a small additive constant. Consequently, any estimator approximating Kolmogorov complexity should assign values clustered near *n* for most inputs. High concentration in the upper range of the distribution is, therefore, theoretically expected and does not, by itself, indicate poor sensitivity.

The objective of sensitivity diagnostics in this study is not to verify adherence to a specific theoretical distribution of complexity values. Rather, it is to ensure that the estimator induces sufficient ordinal resolution for meaningful application of rank-based correlation measures such as Kendall’s τ and Spearman’s ρ. These coefficients depend only on relative ordering and are primarily affected by the prevalence of ties.

Within this framework, we assess sensitivity using two structural diagnostics that quantify ordinal resolution. Both are interpreted in light of the theoretical expectation that only a small fraction of strings can be significantly compressible.

Proportion of non-tied pairs.

For a domain of *N* strings, there are N2 unordered pairs. Let $n_{v}$ denote the number of strings assigned value *v* by the estimator. The proportion of non-tied pairs is

(10) πnon−tied=1−∑vnv2N2.

This quantity measures the fraction of pairwise comparisons that result in strict orderings. Low values indicate limited ordinal resolution, directly constraining the interpretability of Kendall’s τ. We adopt a threshold of $\pi_{\mathrm{non}-\mathrm{tied}}\geq 0.1$, ensuring that at least 10% of all possible pairs are distinguishable.

Effective number of distinct values.

Let $p_{i}$ denote the proportion of strings assigned the *i*-th distinct value. The Shannon entropy of the induced value distribution is

(11) $$H=-\sum_{i}p_{i}\log_{2}p_{i}.$$

From this entropy, we compute the effective number of distinct values:

(12) $$N_{\mathrm{eff}}=2^{H}.$$

This measure captures how many “informationally distinct” levels the estimator provides, penalizing distributions where one value dominates. We require $N_{\mathrm{eff}}\geq 5$ for domains of size |D|≥100. This threshold corresponds to requiring that the estimator can discriminate at least five distinct levels of complexity—a modest demand given that the theory predicts a small but non-negligible tail of compressible strings.

Interpretation within AIT constraints.

Both diagnostics are interpreted relative to the theoretical expectation that only a small fraction of strings can be significantly compressible. The thresholds are deliberately modest: they exclude only those configurations that are so insensitive as to render rank correlation uninterpretable, while remaining compatible with the concentration of complexity values predicted by AIT. Estimators that satisfy both criteria are included in the correlation analysis described in Section 3.5; those that do not are excluded from rank-based comparisons, and this exclusion is itself reported as a diagnostic finding about their limited ordinal resolution in the domain under study.

**Remark** **2.** *The thresholds $\pi_{\mathrm{non}-\mathrm{tied}}\geq 0.1$ and $N_{\mathrm{eff}}\geq 5$ should be understood as conservative lower bounds for ordinal interpretability rather than as claims of optimality. With fewer than 10% non-tied pairs, Kendall’s τ becomes dominated by ties and its interpretation increasingly unstable. The requirement $N_{\mathrm{eff}}\geq 5$ is methodological rather than theoretical: it ensures that the ranking induced by the estimator contains more than a trivial stratification, while remaining modest relative to the domain size. Choosing more demanding thresholds would exclude additional estimators (e.g., those with lower variability) but would not alter the main conclusions of this study. A quantitative sensitivity analysis varying the thresholds, together with the derivation of fully data-driven criteria from statistical learning or information-theoretic principles, is a natural extension and is recorded as future work.*

### 3.5. Rank-Based Correlation Analysis

When comparing different estimation methods for algorithmic complexity, the central question is whether both methods preserve a similar relative ordering of strings according to their estimated complexity values. Because algorithmic complexity is incomputable and defined only up to an additive constant, empirical comparisons between approximations should prioritize their ability to produce consistent rankings rather than exact numerical agreement.

In this context, rank-based correlation coefficients provide a principled and non-parametric framework for assessing agreement between complexity estimators. We employ two complementary measures: Spearman’s rank correlation coefficient (ρ) and Kendall’s tau (τ). Both are symmetric measures (the correlation between *A* and *B* does not depend on which estimator is considered first) in the sense that the resulting score reflects a genuinely mutual notion of agreement rather than a directional comparison. Unlike Pearson’s correlation, which assumes a linear relationship and requires interval-scale data, both Spearman and Kendall rely solely on ordinal information, making them robust to differences in scale, non-linear monotonic relationships, and outliers.

#### 3.5.1. Spearman’s Rank Correlation Coefficient

Spearman’s ρ measures the strength of monotonic association between two variables by applying Pearson’s correlation to the ranks of the data. Given a set of *n* paired observations $\left(x_{i},y_{i}\right)$, let $R_{X}\left(i\right)$ and $R_{Y}\left(i\right)$ denote the ranks of $x_{i}$ and $y_{i}$ respectively. When there are no ties, Spearman’s ρ can be computed using the simplified formula:

(13) $$\rho =1-\frac{6\sum_{i=1}^{n}d_{i}^{2}}{n(n^{2}-1)},$$

where $d_{i}=R_{X}\left(i\right)-R_{Y}\left(i\right)$. When ties are present, we use the general definition based on covariance:

(14) $$\rho =\frac{\mathrm{Cov}(R_{X},R_{Y})}{\sigma_{R_{X}}\sigma_{R_{Y}}}.$$

This formulation reveals an important property for our analysis: if one variable is constant—and, therefore, all its ranks are equal—its standard deviation becomes zero and ρ is undefined. More generally, when variability is extremely low, the coefficient becomes unstable and difficult to interpret.

#### 3.5.2. Kendall’s Tau

While Spearman’s ρ captures global monotonic agreement, Kendall’s τ focuses on pairwise concordance. For a set of *n* observations, Kendall’s τ is defined as:

(15) $$\tau =\frac{\left(\mathrm{number}\;\mathrm{of}\;\mathrm{concordant}\;\mathrm{pairs})-(\mathrm{number}\;\mathrm{of}\;\mathrm{discordant}\;\mathrm{pairs}\right)}{n(n-1)/2},$$

with appropriate adjustments when ties are present. A pair of observations $\left(x_{i},y_{i}\right)$ and $\left(x_{j},y_{j}\right)$ is concordant if the ranks agree in order (both $x_{i}>x_{j}$ and $y_{i}>y_{j}$, or both $x_{i}<x_{j}$ and $y_{i}<y_{j}$); it is discordant if they disagree.

Spearman’s ρ is more sensitive to the magnitude of rank differences, whereas Kendall’s τ is more sensitive to exact pairwise agreement. Using both measures provides a more complete picture of estimator consistency, distinguishing between strong global monotonicity and fine-grained pairwise agreement.

Kendall’s τ is computed using the τ−b variant implemented in scipy.stats.kendalltau, which includes explicit correction terms for ties in both variables. Spearman’s ρ is computed using scipy.stats.spearmanr, where ranks are assigned using the standard average (mid-rank) method in the presence of ties [21] These implementations ensure that ties are handled consistently and that the resulting coefficients remain well-defined even when estimators produce clustered values.

#### 3.5.3. Statistical Significance

Statistical significance of the reported correlation coefficients is assessed using the *p*-values provided by standard implementations (SciPy’s spearmanr and kendalltau functions). These implementations compute *p*-values based on asymptotic approximations: for Spearman’s ρ, a *t*-distribution with n−2 degrees of freedom is used; for Kendall’s τ, a normal approximation is employed.

Given the large domain sizes in the regimes of our study, statistical significance is expected even for moderate correlations. Therefore, effect size (magnitude of ρ and τ) rather than *p*-value is used as the primary interpretative criterion.

#### 3.5.4. Conditions for Meaningful Rank Correlation

As discussed in Section 3.4, the usefulness of rank correlation is critically dependent on the estimator sensitivity. When estimates lack sufficient variability, rank correlation becomes unstable and uninformative. In our analysis, we, therefore, apply rank correlation only in regimes where the variability condition is satisfied.

For BDM estimates, we have verified through the validation procedure in Section 3.3.1 that they maintain adequate sensitivity across all string lengths considered in our comparative analysis. For compression-based estimators, we first perform the diagnostic analysis described in Section 3.4 to assess sensitivity; where variability is adequate, we include them in the rank correlation analysis; where it is not, we report this finding and exclude them from quantitative comparisons.

### 3.6. Control Estimator: BDM_Id_

A fundamental challenge in interpreting correlations between complexity estimators is distinguishing genuine structural agreement from agreement driven by lower-level features common to both methods. In our context, BDM and compression-based estimators may correlate simply because both are sensitive to block repetitions and string length, rather than because they share a deeper algorithmic perspective.

To address this challenge, we introduce a control estimator that we denote BDM_Id_. The subscript “Id” refers to the simplest possible reference machine: the identity machine, which halts immediately and outputs its input unchanged. In such a machine, the shortest program that produces a block *b* is precisely *b* itself, so the complexity assigned to any block is simply its length |b|.

Substituting this into the BDM formula yields:

(16) $${\mathrm{BDM}}_{\mathrm{Id}}\left(s\right)=\sum_{i}\left[|b_{i}|+\log_{2}\left(n_{i}\right)\right],$$

where $|b_{i}|$ denotes the length of block $b_{i}$ and $n_{i}$ its multiplicity in the decomposition. This control estimator is not intended to represent a neutral or structure-free estimator. Rather, it preserves the full decomposition structure of BDM—including block boundaries, block sizes, and the logarithmic penalty for repetitions—while removing the algorithmic information contributed by CTM. In essence, it represents what BDM would assign if the reference machine had no compression capabilities whatsoever.

This control serves three crucial purposes in our analysis:

- Baseline for BDM: By comparing BDM estimates with BDM_Id_, we can quantify how much of BDM’s complexity assignments are attributable to the intrinsic information in CTM vs. the structural features captured by block decomposition and repetition counting.
- Baseline for compression: By comparing compression-based estimates with BDM_Id_, we can assess whether compressors are primarily detecting the same low-level structural features (block boundaries, repetitions) or whether they capture additional regularities.
- Context for cross-paradigm correlation: When evaluating the correlation between BDM and a compression-based estimator, we compare it against the correlations of each with BDM_Id_. If ρ(BDM,Comp) is not significantly larger than both $\rho (\mathrm{BDM},{\mathrm{BDM}}_{\mathrm{Id}})$ and $\rho (\mathrm{Comp},{\mathrm{BDM}}_{\mathrm{Id}})$, then the observed agreement may be largely attributable to shared sensitivity to block structure and repetition, rather than to deeper algorithmic regularities.

In this way, BDM_Id_ provides a rigorous baseline against which to interpret the correlations between target estimators. It allows us to move beyond simply reporting whether two estimators agree, toward understanding the nature and source of their agreement.

### 3.7. Experimental Setup

This section details the concrete implementation of the estimators and domains used in our comparative analysis, providing the necessary information for reproducibility.

#### 3.7.1. Computational Models for CTM

We consider three reference computational models for CTM, each proposed in the literature:

- TM: Classical Turing machine model as described in [8].
- IMP1b: A model based on instruction languages, introduced in [9].
- IMP2: An alternative instruction-based model, also from [10].

For each model M∈{TM,IMP1b,IMP2}, we have precomputed CTM tables covering all strings of length up to $b_{\max}=12$ bits. These tables provide the base complexity values for BDM.

#### 3.7.2. BDM Configurations

For each model *M*, we have precomputed CTM tables covering all strings of length up to a model-specific maximum:

- TM: $b_{\max}=12$ bits [8].
- IMP1b: $b_{\max}=8$ bits [9].
- IMP2: $b_{\max}=6$ bits [10].

For each model *M*, BDM estimates are computed using block sizes $b\leq b_{\max}\left(M\right)$. Following the validation procedure in Section 3.3.1, we select the maximum block size $b_{\max}\left(M\right)$ for subsequent analyses with that model, as it maximizes the information available from CTM while maintaining consistency with finer-grained decompositions. Estimates are denoted as ${\mathrm{BDM}}_{\mathrm{TM}}^{\left(12\right)}$, ${\mathrm{BDM}}_{\mathrm{IMP}1b}^{\left(8\right)}$, and ${\mathrm{BDM}}_{\mathrm{IMP}2}^{\left(6\right)}$, where the superscript indicates the block size used.

For strings whose length is not a multiple of $b_{\max}\left(M\right)$, the final incomplete block is treated as is (no padding), since CTM values are available for all lengths $\leq b_{\max}\left(M\right)$.

#### 3.7.3. Compression-Based Estimators

We consider two compression-based estimators:

Standard Deflate (ZLIB implementation).

We selected Deflate [22] (the algorithm used in gzip) as a representative general-purpose compressor. Deflate combines LZ77 [23] sliding-window compression with Huffman coding. We use the Python 2.3.3 ZLIB implementation of Deflate [24]. For this estimator, we adopt the byte-per-bit input format: each bit of the original binary string is encoded as a full byte (ASCII ‘0’ or ‘1’). This choice yields substantially greater variability in compressed lengths compared to compact byte packing, which produces nearly constant outputs for strings up to 35 bits. Compressibility is measured as the size in bytes of the compressed representation.

Custom bit-level LZ77 implementation (${\mathrm{LZ}77}_{\mathrm{bit}}^{\left(b\right)}$).

To gain finer control over the compression process and better align it with BDM’s capabilities, we implemented a custom LZ77-based compressor that operates directly at the bit level. Like LZSS [25], it uses a 1-bit flag to distinguish between literals and (offset, length) references. However, unlike standard LZSS which only emits a reference when it reduces the output size, our implementation replaces matches unconditionally whenever a match is found, regardless of whether the reference is shorter than the literal. This design choice prioritizes estimator sensitivity over compression efficiency, aligning with our focus on ordinal resolution rather than absolute compressibility. We denote each configuration as ${\mathrm{LZ}77}_{\mathrm{bit}}^{\left(b\right)}$, where the superscript *b* indicates the maximum match length allowed (set to match BDM’s block size $b_{\max}\left(M\right)$). This implementation offers several advantages:

- Bit-level operation: By working directly on bits rather than bytes, the algorithm avoids the arbitrary byte boundaries that can obscure structural patterns in binary data, increasing its sensitivity to regularities at the bit level.
- Controlled match length: The maximum match length is set to $b_{\max}\left(M\right)$, preventing the compressor from exploiting repetitions longer than those detectable by BDM as repeated blocks. This ensures a more balanced comparison between the two approaches.
- Window size: The sliding window is configured to be sufficiently large to cover the entire string under compression, ensuring that all potential repetitions within the string can be detected. At the same time, it is not set unnecessarily larger than the string length, as larger windows would increase the bit cost of encoding offsets in back-references without providing additional compression benefit for strings of this size.
- No Huffman coding: Compression reflects only LZ77 parsing, making estimates more directly comparable to BDM’s treatment of repetitions.
- Enhanced sensitivity: Preliminary experiments indicate that this bit-level implementation exhibits greater variability in compressed lengths, particularly for the relatively short strings that are the focus of our study, making it more suitable for rank-based correlation analysis.

#### 3.7.4. String Domains

We consider binary strings across two main domains:

Exhaustive domain (12≤L≤25): For each length *L* from 12 to 25 bits, we include all strings of that length. With block size b=12, this range lies entirely within the positionally unambiguous regime (L<36) for the TM model. For the lower-resolution models IMP1b ($b_{\max}=8$) and IMP2 ($b_{\max}=6$), part of this domain falls into the positionally ambiguous regime.

Random samples (500≤L≤1500): For longer strings, we generate random samples with the following parameters, chosen to balance statistical power, computational cost, and representativeness:

- Length range: Strings of lengths from 500 to 1500 bits. This range ensures that compression-based estimators exhibit sufficient variability while remaining computationally feasible.
- Sampling granularity: Samples are drawn at 100-bit intervals, providing adequate resolution to detect trends without excessive sampling points.
- Total sample size: 1,000,000 strings, guaranteeing standard errors below 0.001 for correlation estimates.
- Allocation strategy: A hybrid balanced-proportional allocation is used: 1000 strings per length as a baseline ensure per-length statistical power, with the remaining capacity distributed proportionally to the length, reflecting the growing space of possible strings.

#### 3.7.5. Experimental Procedures

For each pair of estimators $\left({\bar{K}}_{1},{\bar{K}}_{2}\right)$ where ${\bar{K}}_{1}$ is a BDM variant and ${\bar{K}}_{2}$ a compression-based estimator (ZLIB or ${\mathrm{LZ}77}_{\mathrm{bit}}^{\left(b\right)}$), we obtain correlation measures across two dimensions:

- Per-length correlations: Computed separately for each string length *L* in the exhaustive domain (12≤L≤25) and for each sampled length in the random domain (500≤L≤1500). This fine-grained analysis controls for trivial length effects and reveals structural consistency at each length.
- Global correlations: Computed across all strings in the exhaustive domain (pooling all lengths 12 to 25) and across all random samples. This aggregated perspective captures overall trends but must be interpreted in light of the control estimator to identify spurious correlations driven primarily by string length.

For each length, we compute:

- Spearman’s ρ and Kendall’s τ between ${\bar{K}}_{1}$ and ${\bar{K}}_{2}$, for each domain.
- Spearman’s ρ and Kendall’s τ between ${\bar{K}}_{2}$ and the control estimator ${\mathrm{BDM}}_{\mathrm{Id}}$, for the random samples domains. For each model *M*, we compute the control estimator using the same block size $b_{\max}\left(M\right)$ as the corresponding BDM configuration.

All correlation coefficients use SciPy’s implementations with asymptotic *p*-values. The sensitivity diagnostics from Section 3.4 are applied to each compression-based estimators for each correlation computation; configurations failing the criteria ($\pi_{\mathrm{non}\text{-}\mathrm{tied}}\geq 0.1$, $N_{\mathrm{eff}}\geq 5$) are reported but excluded from main comparative analyses.

This dual-domain, dual-perspective approach allows us to: (i) evaluate fine-grained structural consistency in the region where BDM is most reliable (12–25 bits), (ii) validate the stability of correlations for longer strings through random sampling, and (iii) distinguish genuine agreement from spurious correlations using the control estimator.

## 4. Results

We present our results in four parts. First, we examine the internal stability of BDM across different block sizes, confirming its reliability as a bridging estimator. Second, we analyze cross-paradigm correlations in the exhaustive domain of short strings (12≤L≤25 bits). Third, we examine correlations in random samples of long strings (500≤L≤1500 bits). Fourth, we use the control estimator BDM_Id_ to distinguish genuine structural agreement from spurious correlations driven by string length or decompositions effects. Throughout, we compare results across the three computational models (TM, IMP1b, IMP2) and two compression estimators (ZLIB and ${\mathrm{LZ}77}_{\mathrm{bit}}$).

### 4.1. BDM Internal Stability

**Figure 1:**
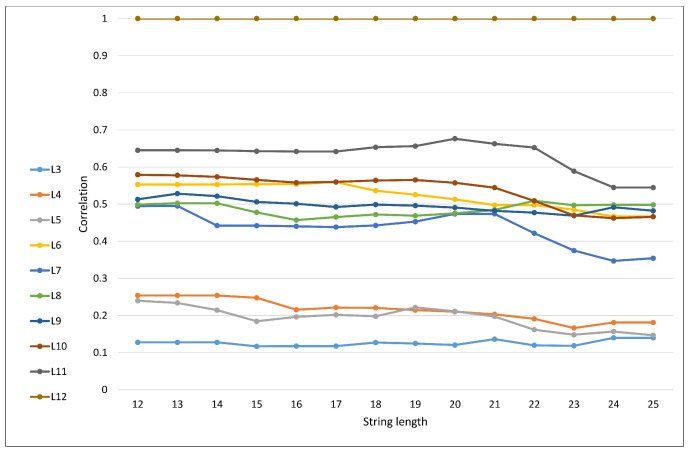
Internal stability of BDM estimates for the TM model ($b_{\max}=12$). Each line shows Spearman’s ρ between estimates using the maximum block size and a smaller block size *b*, as indicated in the legend.

**Figure 2:**
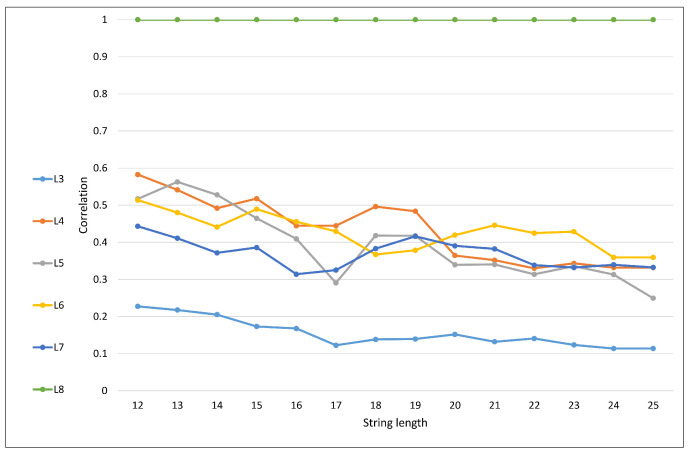
Internal stability of BDM estimates for the IMP1b model ($b_{\max}=8$). Each line shows Spearman’s ρ between estimates using the maximum block size and a smaller block size *b*, as indicated in the legend.

**Figure 3:**
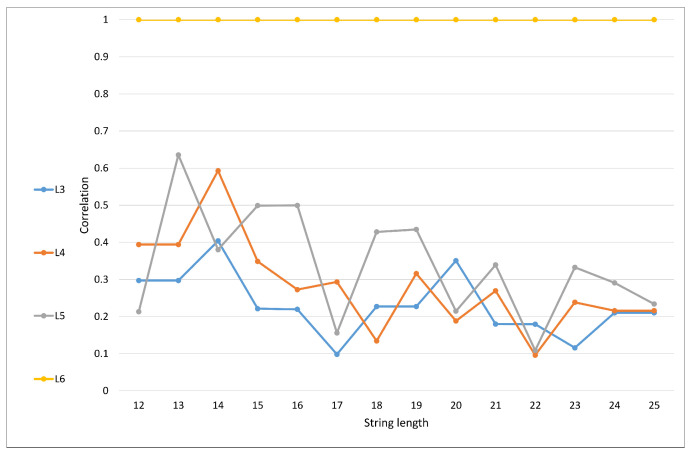
Internal stability of BDM estimates for the IMP2 model ($b_{\max}=6$). Each line shows Spearman’s ρ between estimates using the maximum block size and a smaller block size *b*, as indicated in the legend.

Before using BDM as a bridge between paradigms, we first evaluate the internal stability of BDM as the block size approaches the maximum available value $b_{\max}$ for each model. Figure 1, Figure 2 and Figure 3 present Spearman’s ρ as a function of string length between estimates using the reference configuration (maximum block size $b_{\max}$) and smaller block sizes $b<b_{\max}$ for each computational model, computed across all strings of length 12≤L≤25. The legends identify each curve by the corresponding block size *b* (labeled $L_{b}$). The complete numerical results are provided in Appendix A (Table A1, Table A2 and Table A3).

Figure 1 shows correlations between ${\mathrm{BDM}}_{\mathrm{TM}}^{\left(12\right)}$ and ${\mathrm{BDM}}_{\mathrm{TM}}^{\left(b\right)}$. The expected monotonic increase with block size is clearly visible, with correlations approaching 1.0 as *b* approaches $b_{\max}=12$. The curves remain largely parallel across the length range, indicating stability with respect to string length.

Figure 2 presents an analogous analysis for the IMP1b model ($b_{\max}=8$). While correlations increase on average with block size, the pattern is less regular than for TM. Values for b=7 range from 0.3 to 0.45 and do not consistently exceed those for smaller blocks, showing that convergence is only partial in this lower-resolution model.

Figure 3 shows results for the IMP2 model ($b_{\max}=6$). Despite considerable fluctuations, a discernible increasing trend with block size is observable. Correlations with b=5 exceed those for smaller blocks in approximately 70% of cases.

Examining the three figures together reveals several consistent patterns:

- Monotonic increase with block size: TM exhibits a clear and consistent increase across all lengths. IMP2 shows a discernible trend despite considerable fluctuations. For IMP1b, the pattern is weaker: values for b=7 often fall below those for smaller blocks, indicating only partial convergence.
- Stability across lengths: For each block size *b*, correlations remain reasonably stable across the length range, with no systematic monotonic degradation as *L* increases. This indicates that BDM’s ordinal rankings are robust to string length within each model’s domain. The degree of stability varies by model: TM exhibits the highest consistency, while IMP1b and IMP2 show greater variability, with IMP2 displaying the most pronounced fluctuations.
- Model-specific resolution: The magnitude of correlations for a given block size relative to $b_{\max}$ reflects the underlying resolution of each computational model. TM ($b_{\max}=12$) achieves the highest correlations for intermediate block sizes, followed by IMP1b ($b_{\max}=8$), while IMP2 ($b_{\max}=6$) shows the lowest values, particularly for b=3 and b=4.

These results indicate that the rankings induced by BDM tend to converge as the block size approaches $b_{\max}$, supporting the use of this configuration as the highest-resolution estimator available for each model. The stability observed provides confidence that any differences in cross-paradigm correlations between models reflect genuine differences in their ability to capture structure rather than artifacts of the BDM decomposition.

### 4.2. Correlations in the Exhaustive Domain

We now examine correlations between BDM estimates and compression-based estimators across all strings of lengths 12≤L≤25 bits. This domain lies entirely within the positionally unambiguous regime for TM (L<36), while for IMP1b and IMP2 it includes the transition to the positionally ambiguous regime (L≥24 and L≥18, respectively).

Following the sensitivity diagnostics described in Section 3.4, ZLIB was found to meet the variability criteria for all lengths except the shortest (L=12 and L=13). These lengths are, therefore, excluded from the correlation analyses. For LZ77, all lengths satisfy the criteria. Detailed per-length results, including sensitivity indicators, are provided in Appendix B.

#### 4.2.1. Per-Length Analysis

**Figure 4:**
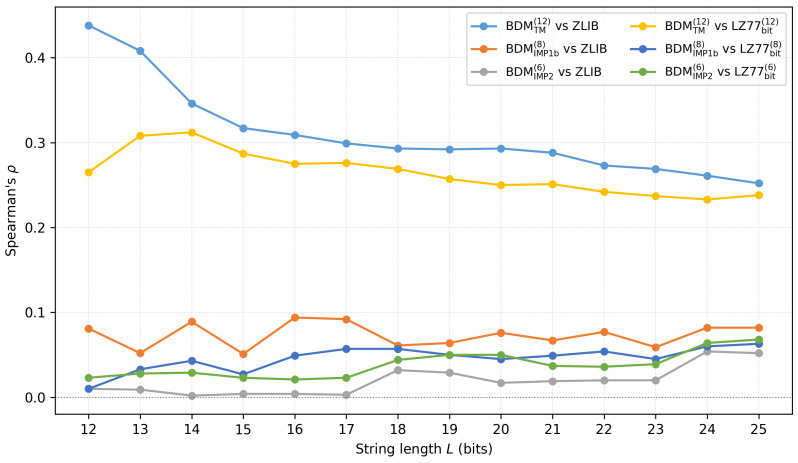
Spearman’s ρ as a function of string length for each model and compressor, in the exhaustive domain.

Figure 4 shows Spearman’s ρ as a function of string length for each model and compressor; the corresponding Kendall’s τ values (provided in Appendix B) exhibit the same patterns and are approximately 0.7 times the Spearman correlations, confirming that the observed trends are robust to the choice of rank correlation measure. Detailed tables can be found in Appendix B.

Several consistent patterns emerge:

- TM ($b_{\max}=12$): Correlations range from 0.25 to 0.44, with a gradual decline as string length increases. ZLIB consistently yields higher correlations than ${\mathrm{LZ}77}_{\mathrm{bit}}$ across all lengths, with the largest differences observed for shorter strings (L≤14). The same pattern holds for Kendall’s τ, with values approximately 30% lower than Spearman’s ρ.
- IMP1b ($b_{\max}=8$): Correlations are substantially lower, typically between 0.05 and 0.09 for Spearman and between 0.03 and 0.07 for Kendall. ZLIB generally outperforms ${\mathrm{LZ}77}_{\mathrm{bit}}$, though the differences are smaller than for TM, and both compressors yield similarly low values. No clear trend with length is observable, and the transition to the positionally ambiguous regime (L≥24) does not produce a noticeable change.
- IMP2 ($b_{\max}=6$): Correlations are very low, with Spearman ranging from 0.002 to 0.068 and Kendall from 0.001 to 0.048. A slight increase is observed in the transition to the positionally ambiguous regime (L≥18) for both measures. Notably, ${\mathrm{LZ}77}_{\mathrm{bit}}$ yields marginally higher values than ZLIB across all lengths, though both remain below 0.07 for Spearman and below 0.05 for Kendall in most cases.

Most of these correlations are statistically significant (p≪0.001), with the exception of a few isolated cases (e.g., L=12 for IMP1b and IMP2). However, their low magnitudes reveal limited practical agreement.

A per-length overlay of the control estimator ${\mathrm{BDM}}_{\mathrm{Id}}^{\left(b\right)}$ is not included in Figure 4 because, for L<3b, the number of distinct values produced by ${\mathrm{BDM}}_{\mathrm{Id}}^{\left(b\right)}$ is extremely low, at most two, which violates the sensitivity criteria of Section 3.4 and renders per-length rank correlations uninformative. Moreover, since per-length correlations are computed separately for each fixed length, the confounding effect of string length is already controlled by construction, making the comparison with the control estimator less informative than in global correlations. The corresponding pooled-domain (global) comparison is reported in Table 1.

#### 4.2.2. Global Correlations and Control Comparison

Table 1 presents global Spearman and Kendall correlations across all strings in the exhaustive domain (*N* = 67,104,768), together with correlations against the control estimator BDM_Id_.

The interpretation of these results by model is as follows:

- TM ($b_{\max}=12$): The correlation between BDM and ZLIB (ρ=0.392, τ=0.291) is modest, while the control estimator correlates at ρ=0.316 (τ=0.273). For ${\mathrm{LZ}77}_{\mathrm{bit}}$, correlations are similar (ρ=0.400, τ=0.284), as well as for the control (ρ=0.352, τ=0.291). This indicates that approximately 88% of the Spearman correlation and essentially all of the Kendall correlation can be explained by the block decomposition structure alone, as the control estimator slightly exceeds BDM in Kendall (τ=0.291 vs. 0.284).
- IMP1b ($b_{\max}=8$): Remarkably, the control estimator outperforms BDM in all correlations, despite having only 16 distinct values compared to BDM’s 11.8 million. This indicates that incorporating CTM information actually reduces agreement with compression-based estimators. The higher correlation of the control estimator suggests that the block structure alone aligns better with what the compressors detect.
- IMP2 ($b_{\max}=6$): The effect is even more pronounced. BDM correlates weakly with both compression estimators (ρ≤0.235) while the control, with just 24 distinct values, achieves substantially higher correlations (ρ≥0.315, up to 0.403). The negative gaps confirm that, for this low-resolution model, CTM information actively hinders the agreement with compression.

#### 4.2.3. Summary of Exhaustive Domain Results

- Control estimator reveals spurious agreement: Across all models, a substantial portion of the correlation between BDM and compression is explained by BDM_Id_, indicating that block-level features and length effects dominate the apparent agreement.
- CTM can be counterproductive: For IMP1b and IMP2, BDM correlates *less* with compression than the control does. This suggests that when CTM resolution is limited, the algorithmic information it provides is not only unhelpful but actively diverges from the structure captured by compressors.
- ZLIB vs. ${\mathrm{LZ}77}_{\mathrm{bit}}$: Despite producing more distinct values, the custom bit-level compressor does not consistently improve correlation with BDM. Its higher raw global correlations are matched by equally higher control correlations, leaving the net contribution of CTM unchanged or reduced.

### 4.3. Correlations in Random Samples

To assess whether the patterns observed in the exhaustive domain extend to longer strings, we analyzed random samples of lengths 500≤L≤1500 bits. For the TM model, the highest-resolution model and the one most likely to exhibit any detectable signal, two independent replicates were generated to verify stability. For this model, both compressors satisfied the sensitivity criteria for all lengths in the random sample domain.

Note that for the TM model the entire range 500≤L≤1500 lies within the positionally ambiguous regime (L≥3b). Therefore, the per-length analysis below also constitutes a first empirical view of ${\mathrm{BDM}}_{\mathrm{TM}}^{\left(12\right)}$ in that regime.

For these experiments, the custom bit-level compressor ${\mathrm{LZ}77}_{\mathrm{bit}}$ was configured with a window size of 11 bits. This differs from the exhaustive domain, where a smaller window was used to maintain comparability with BDM’s block size; here, the window size is chosen to be sufficient to cover the entire string length, ensuring that the compressor can detect all potential repetitions within each string.

#### 4.3.1. Per-Length Analysis

**Figure 5:**
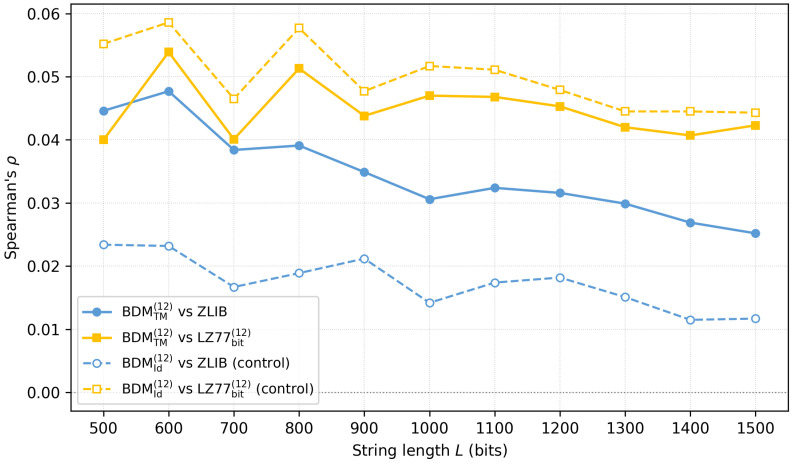
Spearman’s ρ vs. string length for the TM model in the random sample domain. Solid lines: ${\mathrm{BDM}}_{\mathrm{TM}}^{\left(12\right)}$ vs. each compressor. Dashed lines of the same color: control correlation ${\mathrm{BDM}}_{\mathrm{Id}}^{\left(12\right)}$ vs. the same compressor.

Figure 5 shows Spearman’s ρ as a function of string length for each compressor and BDM estimates for the TM model; the corresponding Kendall’s τ values are provided in Appendix C. The per-length correlations obtained from two independent replicates were highly consistent (differences below 0.005 in all cases). Detailed results from the first replicate are provided in Appendix C; the second replicate yielded nearly identical patterns and is omitted for brevity. The main observations are as follows:

- For ZLIB, all correlations are extremely low: Spearman’s ρ ranges from 0.025 to 0.048, and Kendall’s τ from 0.017 to 0.033. No systematic trend with length is observable.
- For ${\mathrm{LZ}77}_{\mathrm{bit}}$, correlations are slightly higher but remain modest: Spearman’s ρ ranges from 0.040 to 0.054, and Kendall’s τ from 0.027 to 0.036.
- The control estimator BDM_Id_ yields correlations of similar magnitude: for ZLIB, ρ ranges from 0.012 to 0.023; for ${\mathrm{LZ}77}_{\mathrm{bit}}$, it ranges from 0.044 to 0.058. Notably, for ${\mathrm{LZ}77}_{\mathrm{bit}}$, the control often exceeds BDM itself, indicating that even the weak signal observed is attributable to block structure and length effects rather than algorithmic information.
- Both compressors exhibit no improvement in agreement at longer lengths; values fluctuate near zero across the entire range.

#### 4.3.2. Global Correlations

While per-length correlations are near zero, pooling all samples across lengths reveals a dramatically different picture. Table 2 presents global Spearman and Kendall correlations computed over the entire set of *N* = 1,000,000 strings spanning lengths 500 to 1500 bits, for both ZLIB and ${\mathrm{LZ}77}_{\mathrm{bit}}^{\left(12\right)}$, together with the cardinality of each estimator.

The global correlations are strikingly high: Spearman’s ρ=0.990 across all four comparisons, and Kendall’s τ ranges from 0.903 to 0.917. At first glance, these values might suggest strong agreement between the estimators. However, a closer examination reveals that this apparent concordance is entirely spurious, driven by three key observations:

- Identical Spearman correlations despite vastly different cardinalities: ${\mathrm{BDM}}_{\mathrm{TM}}^{\left(12\right)}$ assigns a unique value to every string in the sample (|BDM|=1,000,000), while ZLIB produces only 405 distinct values and ${\mathrm{LZ}77}_{\mathrm{bit}}^{\left(12\right)}$ only 1747. Yet all Spearman correlations are identical (ρ=0.990). This can only occur if the correlation is dominated by a single factor common to all estimators: the monotonic relationship with string length.
- Control estimator matches or exceeds BDM: ${\mathrm{BDM}}_{\mathrm{Id}}^{\left(12\right)}$, with a mere 241 distinct values, achieves the same Spearman correlation as ${\mathrm{BDM}}_{\mathrm{TM}}^{\left(12\right)}$ (0.990) and, crucially, yields higher Kendall correlations (0.917 vs. 0.904/0.903). This indicates that the information contributed by CTM does not improve—and in fact slightly degrades—pairwise concordance with compression-based estimators.
- Negligible difference between compressors: Despite ${\mathrm{LZ}77}_{\mathrm{bit}}^{\left(12\right)}$ having over four times the cardinality of ZLIB (1747 vs. 405), both compressors produce nearly identical correlations with BDM and with the control. The enhanced sensitivity of the custom implementation does not translate into better alignment with program-execution-based estimates.

These findings reinforce a central theme of this study: high global correlations can arise from trivial shared dependencies—in this case, the common monotonic relationship with string length—even when per-length agreement is negligible and when estimators differ dramatically in their resolution. The control estimator BDM_Id_ proves essential for diagnosing such spurious concordance: its ability to match or exceed BDM’s correlations demonstrates that no algorithmic information is required to achieve the observed global agreement.

In summary, the random sample analysis yields two complementary and consistent findings:

- Per-length: Correlations are uniformly near zero (ρ<0.05, τ<0.04), indicating the absence of detectable structural agreement under the tested conditions.
- Global: High correlations emerge only when pooling across lengths, but these are entirely explained by the common monotonic relationship with string length, as demonstrated by the control estimator.

Together, these results provide compelling evidence that any apparent agreement between program-execution-based and compression-based estimators is driven by trivial features, primarily string length and block structure, rather than by shared sensitivity to algorithmic complexity.

#### 4.3.3. Note on Other Models

For the lower-resolution models IMP1b and IMP2, which already exhibited near-zero correlations in the exhaustive domain, the long-sample results (available as Supplementary Materials) are even weaker and do not alter any of the conclusions presented here.

For IMP2 in particular, the custom compressor ${\mathrm{LZ}77}_{\mathrm{bit}}^{\left(6\right)}$ with maximum match length $b_{\max}=6$ does not satisfy the per-length sensitivity criteria in the random sample domain, as its limited resolution produces insufficient variability.

### 4.4. Summary of Findings

Our results reveal several key patterns:

- BDM is internally stable: Correlations across block sizes are consistent and monotonic (Appendix A), validating its use as a bridging estimator across all three computational models.
- Weak and inconsistent signal in short strings: In the exhaustive domain (12≤L≤25), TM exhibits modest correlations (ρ≈0.25–0.44) that barely exceed control levels. For IMP1b and IMP2, correlations are essentially null (ρ<0.1) across all lengths.
- Per-length correlations vanish in long strings: In random samples of long strings (500≤L≤1500), per-length correlations for TM are uniformly near zero (Spearman’s ρ≤0.054, Kendall’s τ≤0.036) and indistinguishable from control values. This confirms that no meaningful structural agreement exists at fixed lengths.
- Global correlations are entirely spurious: When aggregating across lengths, high global correlations emerge (ρ=0.990, τ=0.904 for TM vs. LZ77_bit_). However, the control estimator achieves virtually identical values (ρ=0.990, τ=0.918), demonstrating that this apparent agreement is driven exclusively by the common monotonic relationship with string length, not by shared algorithmic information.
- Model resolution is critical: The ordering of correlation magnitudes (TM > IMP1b > IMP2) aligns directly with model resolution ($b_{\max}=12>8>6$). This confirms that BDM’s effectiveness as a bridge depends heavily on the richness of the underlying CTM table.
- LZ77_bit_ offers no structural advantage: Despite its design for enhanced sensitivity and higher cardinality (903 distinct values vs. 405 for ZLIB), the custom bit-level compressor does not improve correlation with BDM over standard ZLIB. Its marginally higher raw correlations are matched by equally higher control correlations, indicating that its advantage is limited to capturing block-level features rather than algorithmic depth.

## 5. Discussion

The results presented above show that compression-based and program-execution-based estimators of algorithmic complexity show limited correspondence across the regimes examined. Per-length correlations are low, and the apparent agreement in global correlations is largely explained by the shared monotonic dependence on string length, as demonstrated by the control estimator BDM_Id_. In this section, we interpret these findings, discuss their practical implications, acknowledge the limitations of our study, and outline directions for future research.

### 5.1. Theoretical Implications

These results should not be read as a criticism of either paradigm; rather, they sharpen our understanding of what each paradigm can and cannot represent in finite, practical regimes. Algorithmic information theory predicts that, for every constant *c*, the overwhelming majority of binary strings of length *n* are incompressible up to *c* bits, so the space of strings on which compressibility differences can carry significant algorithmic information is, by construction, narrow. The empirical picture observed here is consistent with this prediction: outside of trivial length effects, compression-based estimators and CTM/BDM agree only weakly, and most of the residual agreement is captured by the BDM_Id_ control. We interpret this as evidence that compression-based estimators are, in the regimes tested, principally probing statistical regularity (frequency imbalance, local repetition), while CTM/BDM is probing a strict superset of patterns that includes algorithmic regularity, i.e., structure produced by short generative programs even in the absence of statistical redundancy. The fact that BDM_Id_ matches or surpasses BDM under low-resolution CTM tables (IMP1b, IMP2) further suggests that, at insufficient model resolution, the algorithmic information injected by CTM is largely overwhelmed by the block-structural signal common to both paradigms. From the perspective of universal similarity [2] and the interpretation of compressibility as a proxy for *K*, our results argue for treating the equivalence between the two paradigms as a regime-dependent claim rather than as a foundational identity.

### 5.2. Practical Implications

For practitioners, the principal recommendation is to avoid treating compression-based and program-execution-based estimators as interchangeable proxies for algorithmic complexity, particularly when high global correlations across mixed-length corpora are taken as evidence of agreement. Such correlations are largely attributable to the shared monotonic dependence on string length and to block-level structural features captured by BDM_Id_, and so they tend to be present even when no algorithmic signal is shared. A more conservative protocol is to (i) report rank correlations stratified by length wherever possible, (ii) include a structure-only baseline analogous to BDM_Id_ before claiming cross-paradigm agreement, and (iii) interpret correlation magnitude relative to that baseline rather than relative to zero. In applied domains where compression-based proxies are commonly used, such as bioinformatics, time-series analysis, network and pattern classification [2,5,18,19,20], our results suggest that compression remains a reasonable choice when the question of interest is statistical regularity at scale, but that conclusions phrased in terms of algorithmic causality should be supported by program-execution-based estimators or, at minimum, by explicit controls. A hybrid use of both paradigms, as in BDM itself, is consistent with our findings provided that the algorithmic contribution can be empirically distinguished from the structural baseline.

### 5.3. Limitations

Several limitations qualify the scope of our conclusions.

- Choice of compressors. Our findings are conditioned on the specific compressors evaluated: standard Deflate (ZLIB) and a custom bit-level LZ77 variant, both rooted in dictionary-based parsing. Compressors based on different principles, such as PPM or Burrows–Wheeler-based codes, may align differently with CTM/BDM, in particular on strings with statistical structure that LZ-style algorithms cannot exploit at the lengths we consider. Conclusions about the relationship between the two paradigms should, therefore, be read as conditional on the family of compressors actually examined.
- Scope of CTM tables. The program-execution side is limited by the resolution of currently available CTM tables ($b_{\max}\in \left\{6,8,12\right\}$). The qualitative trend that lower-resolution models exhibit weaker cross-paradigm agreement does not preclude the possibility that models with substantially larger $b_{\max}$ would behave differently.
- Data types. The empirical analysis is conducted entirely on synthetic binary strings, drawn either exhaustively in 12≤L≤25 or as random samples in 500≤L≤1500. We do not extend the methodology to non-binary alphabets or real-world corpora such as DNA sequences, images, or graph structures.
- Positionally ambiguous regime. As discussed in Section 3.3.2, the empirical characterization of BDM in the positionally ambiguous regime (L≥3b) for the highest-resolution model (TM, L≥36) remains open. For IMP1b and IMP2, the exhaustive domain only partially samples this regime and does not resolve the transition for TM.

### 5.4. Future Work

The limitations discussed above, together with the directions suggested by our findings, motivate several lines of future work, which we outline below:

- Empirical characterization of the positionally ambiguous regime for TM. The transition L≥3b for the highest-resolution model (b=12, L≥36) is the most direct gap between our results and the conceptual analysis of Section 3.3.2. A controlled study of ${\mathrm{BDM}}_{\mathrm{TM}}^{\left(12\right)}$ vs. compression-based estimators in the range 36≤L≤100, ideally combined with constructions that fix block multisets while permuting block positions, would isolate the contribution of positional information to cross-paradigm disagreement.
- Extension of CTM tables to larger strings. The maximum block sizes available in this study ($b_{\max}\in \left\{6,8,12\right\}$) reflect the current frontier of practical CTM table calculation. New CTM computations for larger spaces would relax the central methodological asymmetry of this comparison and enable direct evaluation of whether the gap in cross-paradigm agreement closes as model resolution increases.
- Validation against labelled generators. Strings produced by Fibonacci words, random walks, automatic sequences, low-state cellular automata, and other algorithmic generators provide a partial ground-truth ordering, at least relative to a class of trivially incompressible random strings, against which both compression-based and program-execution-based rankings can be assessed.
- Compressor diversity. Replicating the analysis with non-LZ compressors, such as context-mixing schemes, PPM, and Burrows–Wheeler transform-based codes, would identify which structural patterns each compressor family shares with CTM/BDM.
- Input representation and compressor configuration sweep. A systematic exploration of bit-packing vs. byte-packing inputs, padding strategies, and compressor parameters would quantify the contribution of representation choices to estimator sensitivity and to cross-paradigm agreement. The byte-per-bit choice adopted here was motivated by sensitivity considerations for short strings.
- Extension to non-binary alphabets and real data. Replicating this comparative methodology on graphs, images, and biological sequences (DNA, protein) would assess the external validity of the conclusions beyond synthetic binary strings, and would connect this framework to the applied domains in which compression-based proxies are most heavily used.
- Positionally aware extensions of BDM. The underestimation effect identified in the positionally ambiguous regime invites a refined BDM formulation that encodes positional information about repeated blocks, for example, via an additional term that accounts for the entropy of block arrangements, at a controlled cost in tractability. Such an extension would also provide a natural benchmark for the empirical study suggested in item 1.
- Data-driven sensitivity thresholds. The thresholds $\pi_{\mathrm{non}-\mathrm{tied}}\geq 0.1$ and $N_{\mathrm{eff}}\geq 5$ used here (cf. Remark 2) are conservative lower bounds. A principled derivation from statistical learning or information-theoretic considerations, supported by a quantitative sweep of threshold values, would further validate the robustness of the criteria.

## 6. Conclusions

This study provides a systematic empirical comparison between two major families of practical approximations to algorithmic complexity: compression-based estimators and program-execution-based estimators. Rather than seeking to validate one approach against the other, we aim to characterize their relationship and the extent to which they capture similar structural features. By introducing a control estimator (BDM_Id_) that isolates block structure from algorithmic information, we established a rigorous baseline for interpreting cross-paradigm correlations.

Our results reveal three main findings. First, cross-paradigm correlations are consistently weak when controlling for string length. In the exhaustive domain of short strings, correlations are modest and often close to the control baseline. In longer strings, per-length correlations vanish almost entirely (ρ<0.05), while high global correlations emerge only when aggregating across lengths, a pattern largely explained by the common monotonic relationship with string length, as demonstrated by the control estimator.

Second, model resolution critically affects BDM’s ability to capture structure. Models with lower $b_{\max}$ (IMP1b, IMP2) yield systematically lower correlations, and for these models, the control estimator BDM_Id_ outperforms BDM itself. This indicates that when CTM information is limited, it fails to improve agreement and, in several cases, slightly reduces it, suggesting that the types of structure captured by each paradigm may differ substantially.

Third, despite its design for enhanced sensitivity, the custom bit-level compressor ${\mathrm{LZ}77}_{\mathrm{bit}}$ offers no structural advantage over standard ZLIB. Its marginally higher raw correlations are matched by equally higher control correlations, suggesting that its benefits are primarily limited to capturing block-level features rather than deeper algorithmic structure.

In summary, this work shows that the empirical agreement between compression-based and program-execution-based estimators of algorithmic complexity is largely attributable to trivial features, namely string length and block structure, rather than to consistent shared sensitivity to algorithmic information. This suggests that the algorithmic structure captured by each paradigm may be fundamentally different in nature, at least within the regimes accessible with current CTM tables and the compressor families considered. Compression excels at detecting statistical regularities, while CTM and BDM offer a pathway to identifying algorithmic causality. The two paradigms are not interchangeable, and understanding their respective strengths and limitations is essential for their appropriate application. The control mechanisms introduced here provide a framework for such comparative assessments, paving the way for more nuanced empirical studies of algorithmic complexity.

## Figures and Tables

**Table 1:** Global Spearman and Kendall correlations in the exhaustive domain (12≤L≤25, N=67,104,768) for all models and compressors, including control comparisons. Cardinalities indicate the number of distinct values produced by each estimator.

Model	Comparison	ρ	τ	$|{\mathrm{Estimator}}_{1}|$	$|{\mathrm{Estimator}}_{2}|$
TM (b=12)	BDM vs. ZLIB	0.392	0.291	315,464	16
	BDM_Id_ vs. ZLIB	0.316	0.273	13	16
	BDM vs. ${\mathrm{LZ}77}_{\mathrm{bit}}^{\left(12\right)}$	0.400	0.284	315,464	34
	BDM_Id_ vs. ${\mathrm{LZ}77}_{\mathrm{bit}}^{\left(12\right)}$	0.352	0.291	13	34
IMP1b (b=8)	BDM vs. ZLIB	0.282	0.207	11,806,307	16
	BDM_Id_ vs. ZLIB	0.324	0.278	16	16
	BDM vs. ${\mathrm{LZ}77}_{\mathrm{bit}}^{\left(8\right)}$	0.307	0.217	11,806,307	31
	BDM_Id_ vs. ${\mathrm{LZ}77}_{\mathrm{bit}}^{\left(8\right)}$	0.376	0.310	16	31
IMP2 (b=6)	BDM vs. ZLIB	0.182	0.133	3,811,226	16
	BDM_Id_ vs. ZLIB	0.315	0.266	24	16
	BDM vs. ${\mathrm{LZ}77}_{\mathrm{bit}}^{\left(6\right)}$	0.235	0.165	3,811,226	29
	BDM_Id_ vs. ${\mathrm{LZ}77}_{\mathrm{bit}}^{\left(6\right)}$	0.403	0.330	24	29

Note: all correlations are statistically significant (p≪0.001). $|{\mathrm{Estimator}}_{1}|$ denotes the cardinality of the first estimator in each comparison (BDM or BDM_Id_).

**Table 2:** Global correlations for TM model in the random sample domain (500≤L≤1500, pooled sample, *N* = 1,000,000).

Comparison	ρ	τ	$|{\mathrm{Estimator}}_{1}|$	$|{\mathrm{Estimator}}_{2}|$
${\mathrm{BDM}}_{\mathrm{TM}}^{\left(12\right)}$ vs. ZLIB	0.990	0.904	1,000,000	405
${\mathrm{BDM}}_{\mathrm{Id}}^{\left(12\right)}$ vs. ZLIB (control)	0.990	0.917	237	405
${\mathrm{BDM}}_{\mathrm{TM}}^{\left(12\right)}$ vs. ${\mathrm{LZ}77}_{\mathrm{bit}}^{\left(12\right)}$	0.990	0.904	1,000,000	903
${\mathrm{BDM}}_{\mathrm{Id}}^{\left(12\right)}$ vs. ${\mathrm{LZ}77}_{\mathrm{bit}}^{\left(12\right)}$ (control)	0.990	0.918	237	903

## Data Availability

The code and experimental data supporting the findings of this study are openly available in a public repository at https://github.com/zoe-leyva-acosta/bdm-vs-compression (accessed on 24 March 2026) for this article, containing the implementation of the custom LZ77 compressor, BDM, the experimental scripts, and the complete results.

## Supplementary Materials

The following supporting information can be downloaded at: https://www.mdpi.com/article/10.3390/e28060601/s1.

## Abbreviations

The following abbreviations are used in this manuscript:

AIT	Algorithmic Information Theory
BDM	Block Decomposition Method
CTM	Coding Theorem Method
LZ77	Lempel-Ziv 77 compression algorithm
ZLIB	ZLIB compression library (implementation of Deflate)

## Appendix A. Detailed Stability Tables for BDM Estimates

The following tables present Spearman correlations between BDM estimates using the maximum block size $b_{\max}$ and smaller block sizes $b<b_{\max}$ for each computational model, computed across all strings of length 12≤L≤25. In each table, columns labeled $L_{b}$ indicate the correlation with the configuration using block size *b*.

**Table A1:** Spearman correlations between ${\mathrm{BDM}}_{\mathrm{TM}}^{\left(12\right)}$ and smaller block sizes.

L	L3	L4	L5	L6	L7	L8	L9	L10	L11	L12
12	0.128	0.254	0.240	0.553	0.495	0.499	0.513	0.579	0.645	1.000
13	0.128	0.254	0.234	0.553	0.495	0.503	0.528	0.578	0.645	1.000
14	0.128	0.254	0.214	0.553	0.442	0.502	0.521	0.574	0.645	1.000
15	0.117	0.248	0.184	0.554	0.442	0.478	0.506	0.565	0.643	1.000
16	0.117	0.215	0.196	0.554	0.440	0.457	0.501	0.558	0.642	1.000
17	0.117	0.221	0.202	0.559	0.438	0.465	0.492	0.560	0.642	1.000
18	0.127	0.220	0.198	0.536	0.443	0.472	0.499	0.564	0.653	1.000
19	0.124	0.214	0.222	0.525	0.453	0.469	0.496	0.565	0.656	1.000
20	0.120	0.210	0.211	0.513	0.474	0.475	0.491	0.557	0.676	1.000
21	0.136	0.203	0.197	0.497	0.474	0.484	0.482	0.544	0.663	1.000
22	0.120	0.191	0.162	0.498	0.422	0.509	0.477	0.509	0.652	1.000
23	0.118	0.166	0.148	0.485	0.375	0.497	0.469	0.470	0.589	1.000
24	0.140	0.181	0.157	0.467	0.347	0.498	0.492	0.462	0.545	1.000
25	0.140	0.181	0.147	0.467	0.354	0.498	0.482	0.466	0.545	1.000

**Table A2:** Spearman correlations between ${\mathrm{BDM}}_{\mathrm{IMP}1b}^{\left(8\right)}$ and smaller block sizes.

L	L3	L4	L5	L6	L7	L8
12	0.227	0.583	0.517	0.514	0.443	1.000
13	0.218	0.541	0.563	0.480	0.411	1.000
14	0.205	0.492	0.528	0.441	0.372	1.000
15	0.173	0.518	0.464	0.490	0.386	1.000
16	0.168	0.445	0.409	0.455	0.314	1.000
17	0.122	0.445	0.291	0.430	0.325	1.000
18	0.138	0.496	0.418	0.367	0.383	1.000
19	0.139	0.484	0.418	0.379	0.416	1.000
20	0.152	0.364	0.339	0.420	0.390	1.000
21	0.132	0.352	0.340	0.446	0.382	1.000
22	0.141	0.330	0.314	0.425	0.338	1.000
23	0.124	0.343	0.336	0.429	0.332	1.000
24	0.114	0.332	0.313	0.359	0.339	1.000
25	0.114	0.332	0.249	0.359	0.332	1.000

**Table A3:** Spearman correlations between ${\mathrm{BDM}}_{\mathrm{IMP}2}^{\left(6\right)}$ and smaller block sizes.

L	L3	L4	L5	L6
12	0.297	0.394	0.213	1.000
13	0.297	0.394	0.636	1.000
14	0.404	0.593	0.380	1.000
15	0.221	0.349	0.499	1.000
16	0.219	0.273	0.500	1.000
17	0.098	0.293	0.156	1.000
18	0.227	0.134	0.428	1.000
19	0.227	0.316	0.435	1.000
20	0.351	0.188	0.215	1.000
21	0.180	0.269	0.339	1.000
22	0.179	0.096	0.109	1.000
23	0.116	0.238	0.333	1.000
24	0.210	0.216	0.291	1.000
25	0.210	0.216	0.234	1.000

## Appendix B. Detailed Correlation Tables for the Exhaustive Domain

The following tables present per-length Spearman and Kendall correlations between BDM estimates and compression-based estimators for strings of length 12≤L≤25. For each table, $|{\mathrm{BDM}}_{M}|$ and |Comp| indicate the number of distinct values produced by each estimator at that length. The column “Sensitive” indicates whether the estimator satisfies the sensitivity criteria defined in Section 3.4 ($\pi_{\mathrm{non}\text{-}\mathrm{tied}}\geq 0.1$, $N_{\mathrm{eff}}\geq 5$). Rows marked as “No” are excluded from the correlation analyses.

### Appendix B.1. TM Model ($b_{\max}=12$)

**Table A4:** Spearman and Kendall correlations between ${\mathrm{BDM}}_{\mathrm{TM}}^{\left(12\right)}$ and ZLIB, with number of distinct values.

*L*	ρ	*p*-Value (ρ)	τ	*p*-Value (τ)	$|{\mathrm{BDM}}_{\mathrm{TM}}^{\left(12\right)}|$	|ZLIB|	Sensitive
12	0.438	$6.75\times 10^{-192}$	0.336	$2.43\times 10^{-184}$	372	8	No
13	0.408	0	0.311	0	372	9	No
14	0.346	0	0.259	0	372	10	Yes
15	0.317	0	0.236	0	1116	10	Yes
16	0.309	0	0.230	0	2232	11	Yes
17	0.299	0	0.221	0	3720	12	Yes
18	0.293	0	0.217	0	7440	12	Yes
19	0.292	0	0.216	0	13,392	13	Yes
20	0.293	0	0.217	0	26,784	14	Yes
21	0.288	0	0.213	0	50,195	14	Yes
22	0.273	0	0.202	0	94,013	15	Yes
23	0.269	0	0.199	0	118,247	16	Yes
24	0.261	0	0.192	0	51,122	16	Yes
25	0.252	0	0.186	0	51,122	16	Yes

**Table A5:** Spearman and Kendall correlations between ${\mathrm{BDM}}_{\mathrm{TM}}^{\left(12\right)}$ and ${\mathrm{LZ}77}_{\mathrm{bit}}^{\left(12\right)}$, with number of distinct values.

*L*	ρ	*p*-Value (ρ)	τ	*p*-Value (τ)	$|{\mathrm{BDM}}_{\mathrm{TM}}^{\left(12\right)}|$	$|{\mathrm{LZ}77}_{\mathrm{bit}}^{\left(12\right)}|$	Sensitive
12	0.265	$1.39\times 10^{-66}$	0.195	$3.01\times 10^{-67}$	372	13	Yes
13	0.308	$5.23\times 10^{-180}$	0.227	$7.69\times 10^{-182}$	372	14	Yes
14	0.312	0	0.228	0	372	15	Yes
15	0.287	0	0.208	0	1116	16	Yes
16	0.275	0	0.198	0	2232	16	Yes
17	0.276	0	0.198	0	3720	17	Yes
18	0.269	0	0.192	0	7440	18	Yes
19	0.257	0	0.183	0	13,392	19	Yes
20	0.250	0	0.178	0	26,784	21	Yes
21	0.251	0	0.178	0	50,195	23	Yes
22	0.242	0	0.171	0	94,013	24	Yes
23	0.237	0	0.167	0	118,247	26	Yes
24	0.233	0	0.164	0	51,122	28	Yes
25	0.238	0	0.167	0	51,122	29	Yes

### Appendix B.2. IMP1b Model ($b_{\max}=8$)

**Table A6:** Spearman and Kendall correlations between ${\mathrm{BDM}}_{\mathrm{IMP}1b}^{\left(8\right)}$ and ZLIB, with number of distinct values.

*L*	ρ	*p*-Value (ρ)	τ	*p*-Value (τ)	$|{\mathrm{BDM}}_{\mathrm{IMP}1b}^{\left(8\right)}|$	|ZLIB|	Sensitive
12	0.081	$1.87\times 10^{-7}$	0.061	$1.30\times 10^{-7}$	4080	8	No
13	0.052	$2.67\times 10^{-6}$	0.039	$2.06\times 10^{-6}$	8160	9	No
14	0.089	$6.71\times 10^{-30}$	0.066	$2.63\times 10^{-30}$	16,320	10	Yes
15	0.051	$3.58\times 10^{-20}$	0.038	$1.11\times 10^{-20}$	32,640	10	Yes
16	0.094	$2.10\times 10^{-129}$	0.069	$6.42\times 10^{-131}$	32,641	11	Yes
17	0.092	$1.25\times 10^{-245}$	0.068	$7.93\times 10^{-248}$	32,641	12	Yes
18	0.061	$3.13\times 10^{-212}$	0.045	$3.79\times 10^{-214}$	130,564	12	Yes
19	0.064	0	0.047	0	261,128	13	Yes
20	0.076	0	0.056	0	522,256	14	Yes
21	0.067	0	0.049	0	1,044,512	14	Yes
22	0.077	0	0.057	0	2,089,024	15	Yes
23	0.059	0	0.043	0	4,178,048	16	Yes
24	0.082	0	0.060	0	3,486,934	16	Yes
25	0.082	0	0.060	0	3,486,934	16	Yes

**Table A7:** Spearman and Kendall correlations between ${\mathrm{BDM}}_{\mathrm{IMP}1b}^{\left(8\right)}$ and ${\mathrm{LZ}77}_{\mathrm{bit}}^{\left(8\right)}$, with number of distinct values.

*L*	ρ	*p*-Value (ρ)	τ	*p*-Value (τ)	$|{\mathrm{BDM}}_{\mathrm{IMP}1b}^{\left(8\right)}|$	$|{\mathrm{LZ}77}_{\mathrm{bit}}^{\left(8\right)}|$	Sensitive
12	0.010	$5.02\times 10^{-1}$	0.007	$5.08\times 10^{-1}$	4080	10	Yes
13	0.033	$2.64\times 10^{-3}$	0.024	$2.49\times 10^{-3}$	8160	10	Yes
14	0.043	$3.35\times 10^{-8}$	0.031	$3.09\times 10^{-8}$	16,320	11	Yes
15	0.027	$9.11\times 10^{-7}$	0.019	$1.07\times 10^{-6}$	32,640	13	Yes
16	0.049	$5.11\times 10^{-36}$	0.035	$4.19\times 10^{-36}$	32,641	14	Yes
17	0.057	$2.91\times 10^{-96}$	0.041	$8.12\times 10^{-97}$	32,641	16	Yes
18	0.057	$3.50\times 10^{-185}$	0.040	$2.90\times 10^{-186}$	130,564	17	Yes
19	0.050	$6.16\times 10^{-289}$	0.035	$2.14\times 10^{-289}$	261,128	17	Yes
20	0.045	0	0.032	0	522,256	18	Yes
21	0.049	0	0.034	0	1,044,512	19	Yes
22	0.054	0	0.038	0	2,089,024	19	Yes
23	0.045	0	0.032	0	4,178,048	21	Yes
24	0.060	0	0.042	0	3,486,934	23	Yes
25	0.063	0	0.044	0	3,486,934	24	Yes

### Appendix B.3. IMP2 Model ($b_{\max}=6$)

**Table A8:** Spearman and Kendall correlations between ${\mathrm{BDM}}_{\mathrm{IMP}2}^{\left(6\right)}$ and ZLIB, with number of distinct values.

*L*	ρ	*p*-Value (ρ)	τ	*p*-Value (τ)	$|{\mathrm{BDM}}_{\mathrm{IMP}2}^{\left(6\right)}|$	|ZLIB|	Sensitive
12	0.010	$5.08\times 10^{-1}$	0.008	$5.07\times 10^{-1}$	2033	8	No
13	0.009	$4.19\times 10^{-1}$	0.007	$4.15\times 10^{-1}$	2033	9	No
14	0.002	$8.11\times 10^{-1}$	0.001	$8.13\times 10^{-1}$	8128	10	Yes
15	0.004	$5.01\times 10^{-1}$	0.003	$5.00\times 10^{-1}$	16,073	10	Yes
16	0.004	$3.63\times 10^{-1}$	0.003	$3.61\times 10^{-1}$	31,797	11	Yes
17	0.003	$3.12\times 10^{-1}$	0.002	$3.08\times 10^{-1}$	60,462	12	Yes
18	0.032	$1.16\times 10^{-59}$	0.023	$1.29\times 10^{-59}$	51,475	12	Yes
19	0.029	$4.58\times 10^{-97}$	0.021	$7.76\times 10^{-97}$	51,475	13	Yes
20	0.017	$3.83\times 10^{-70}$	0.013	$1.79\times 10^{-69}$	195,187	14	Yes
21	0.019	$1.14\times 10^{-163}$	0.014	$5.64\times 10^{-163}$	405,608	14	Yes
22	0.020	0	0.015	0	769,740	15	Yes
23	0.020	0	0.014	0	1,430,718	16	Yes
24	0.054	0	0.039	0	1,068,015	16	Yes
25	0.052	0	0.038	0	1,068,015	16	Yes

## Appendix C. Detailed Correlation Tables for Random Samples (TM Model)

The following tables present per-length Spearman and Kendall correlations for the TM model in the random sample domain (500≤L≤1500 bits). Results correspond to the first replicate; the second replicate yielded nearly identical patterns and is omitted for brevity. For all tables, *N* denotes the number of strings sampled at each length, $|{\mathrm{BDM}}_{\mathrm{TM}}^{\left(12\right)}|$ and |Comp| indicate the number of distinct values produced by each estimator.

**Table A9:** Spearman and Kendall correlations between ${\mathrm{BDM}}_{\mathrm{IMP}2}^{\left(6\right)}$ and ${\mathrm{LZ}77}_{\mathrm{bit}}^{\left(6\right)}$, with number of distinct values.

*L*	ρ	*p*-Value (ρ)	τ	*p*-Value (τ)	$|{\mathrm{BDM}}_{\mathrm{IMP}2}^{\left(6\right)}|$	$|{\mathrm{LZ}77}_{\mathrm{bit}}^{\left(6\right)}|$	Sensitive
12	0.023	$1.34\times 10^{-1}$	0.017	$1.31\times 10^{-1}$	2033	8	Yes
13	0.028	$1.23\times 10^{-2}$	0.020	$1.21\times 10^{-2}$	2033	9	Yes
14	0.029	$2.47\times 10^{-4}$	0.020	$2.43\times 10^{-4}$	8128	10	Yes
15	0.023	$3.85\times 10^{-5}$	0.016	$3.52\times 10^{-5}$	16,073	11	Yes
16	0.021	$4.22\times 10^{-8}$	0.015	$4.10\times 10^{-8}$	31,797	11	Yes
17	0.023	$5.87\times 10^{-17}$	0.016	$6.49\times 10^{-17}$	60,462	12	Yes
18	0.044	$1.63\times 10^{-112}$	0.031	$2.08\times 10^{-112}$	51,475	13	Yes
19	0.050	$1.36\times 10^{-285}$	0.035	$1.28\times 10^{-285}$	51,475	14	Yes
20	0.050	0	0.035	0	195,187	15	Yes
21	0.037	0	0.026	0	405,608	16	Yes
22	0.036	0	0.025	0	769,740	16	Yes
23	0.039	0	0.028	0	1,430,718	17	Yes
24	0.064	0	0.045	0	1,068,015	18	Yes
25	0.068	0	0.048	0	1,068,015	19	Yes

**Table A10:** Spearman and Kendall correlations between ${\mathrm{BDM}}_{\mathrm{TM}}^{\left(12\right)}$ and ZLIB.

*L*	ρ	*p*-Value (ρ)	τ	*p*-Value (τ)	*N*	$|{\mathrm{BDM}}_{\mathrm{TM}}^{\left(12\right)}|$	|ZLIB|
500	0.0446	$1.03\times 10^{-21}$	0.0307	$1.05\times 10^{-21}$	45,954	45,954	36
600	0.0477	$4.23\times 10^{-29}$	0.0327	$4.87\times 10^{-29}$	54,945	54,945	42
700	0.0384	$2.94\times 10^{-22}$	0.0263	$2.67\times 10^{-22}$	63,936	63,936	42
800	0.0391	$4.20\times 10^{-26}$	0.0267	$4.72\times 10^{-26}$	72,927	72,927	47
900	0.0349	$1.55\times 10^{-23}$	0.0238	$1.58\times 10^{-23}$	81,918	81,918	51
1000	0.0306	$2.61\times 10^{-20}$	0.0209	$2.50\times 10^{-20}$	90,909	90,909	55
1100	0.0324	$1.29\times 10^{-24}$	0.0221	$1.30\times 10^{-24}$	99,900	99,900	57
1200	0.0316	$1.80\times 10^{-25}$	0.0215	$1.78\times 10^{-25}$	108,890	108,890	57
1300	0.0299	$1.00\times 10^{-24}$	0.0203	$9.89\times 10^{-25}$	117,881	117,881	61
1400	0.0269	$9.10\times 10^{-22}$	0.0183	$9.55\times 10^{-22}$	126,872	126,872	63
1500	0.0252	$1.79\times 10^{-20}$	0.0171	$1.87\times 10^{-20}$	135,868	135,868	66

### Appendix C.1. ZLIB Compressor

**Table A11:** Spearman and Kendall correlations between ${\mathrm{BDM}}_{\mathrm{Id}}^{\left(12\right)}$ and ZLIB (control).

*L*	ρ	*p*-Value (ρ)	τ	*p*-Value (τ)	*N*	$|{\mathrm{BDM}}_{\mathrm{Id}}^{\left(12\right)}|$	|ZLIB|
500	0.0234	$5.29\times 10^{-7}$	0.0195	$5.31\times 10^{-7}$	45,954	8	36
600	0.0232	$5.33\times 10^{-8}$	0.0192	$5.45\times 10^{-8}$	54,945	9	42
700	0.0167	$2.34\times 10^{-5}$	0.0137	$2.36\times 10^{-5}$	63,936	12	42
800	0.0189	$3.22\times 10^{-7}$	0.0153	$3.33\times 10^{-7}$	72,927	13	47
900	0.0212	$1.35\times 10^{-9}$	0.0169	$1.33\times 10^{-9}$	81,918	16	51
1000	0.0142	$1.78\times 10^{-5}$	0.0112	$1.80\times 10^{-5}$	90,909	23	55
1100	0.0174	$3.74\times 10^{-8}$	0.0135	$3.75\times 10^{-8}$	99,900	21	57
1200	0.0182	$2.10\times 10^{-9}$	0.0139	$2.07\times 10^{-9}$	108,890	26	57
1300	0.0151	$2.00\times 10^{-7}$	0.0115	$1.98\times 10^{-7}$	117,881	30	61
1400	0.0115	$4.25\times 10^{-5}$	0.0086	$4.27\times 10^{-5}$	126,872	37	63
1500	0.0117	$1.70\times 10^{-5}$	0.0087	$1.71\times 10^{-5}$	135,868	43	66

### Appendix C.2. LZ77 Compressor

For these experiments, ${\mathrm{LZ}77}_{\mathrm{bit}}$ was configured with a window size of 11 bits, sufficient to cover the entire string length.

**Table A12:** Spearman and Kendall correlations between ${\mathrm{BDM}}_{\mathrm{TM}}^{\left(12\right)}$ and ${\mathrm{LZ}77}_{\mathrm{bit}}^{\left(12\right)}$.

*L*	ρ	*p*-Value (ρ)	τ	*p*-Value (τ)	*N*	$|{\mathrm{BDM}}_{\mathrm{TM}}^{\left(12\right)}|$	$|{\mathrm{LZ}77}_{\mathrm{bit}}^{\left(12\right)}|$
500	0.0400	$9.55\times 10^{-18}$	0.0270	$1.01\times 10^{-17}$	45,954	45,954	84
600	0.0539	$1.32\times 10^{-36}$	0.0364	$1.29\times 10^{-36}$	54,945	54,945	88
700	0.0401	$3.19\times 10^{-24}$	0.0271	$3.38\times 10^{-24}$	63,936	63,936	93
800	0.0513	$1.09\times 10^{-43}$	0.0346	$9.38\times 10^{-44}$	72,927	72,927	101
900	0.0438	$4.05\times 10^{-36}$	0.0296	$4.10\times 10^{-36}$	81,918	81,918	101
1000	0.0470	$1.22\times 10^{-45}$	0.0317	$1.23\times 10^{-45}$	90,909	90,909	107
1100	0.0468	$1.81\times 10^{-49}$	0.0316	$1.47\times 10^{-49}$	99,900	99,900	112
1200	0.0453	$1.69\times 10^{-50}$	0.0305	$1.78\times 10^{-50}$	108,890	108,890	107
1300	0.0420	$3.13\times 10^{-47}$	0.0283	$3.04\times 10^{-47}$	117,881	117,881	114
1400	0.0407	$1.41\times 10^{-47}$	0.0274	$1.55\times 10^{-47}$	126,872	126,872	112
1500	0.0423	$8.36\times 10^{-55}$	0.0285	$8.41\times 10^{-55}$	135,868	135,868	118

**Table A13:** Spearman and Kendall correlations between ${\mathrm{BDM}}_{\mathrm{Id}}^{\left(12\right)}$ and ${\mathrm{LZ}77}_{\mathrm{bit}}^{\left(12\right)}$ (control).

*L*	ρ	*p*-Value (ρ)	τ	*p*-Value (τ)	*N*	$|{\mathrm{BDM}}_{\mathrm{Id}}^{\left(12\right)}|$	$|{\mathrm{LZ}77}_{\mathrm{bit}}^{\left(12\right)}|$
500	0.0552	$2.61\times 10^{-32}$	0.0453	$2.82\times 10^{-32}$	45,954	8	84
600	0.0586	$5.71\times 10^{-43}$	0.0477	$6.32\times 10^{-43}$	54,945	9	88
700	0.0465	$6.47\times 10^{-32}$	0.0375	$6.67\times 10^{-32}$	63,936	12	93
800	0.0577	$6.68\times 10^{-55}$	0.0461	$6.95\times 10^{-55}$	72,927	13	101
900	0.0477	$1.99\times 10^{-42}$	0.0374	$2.32\times 10^{-42}$	81,918	16	101
1000	0.0517	$7.38\times 10^{-55}$	0.0401	$7.77\times 10^{-55}$	90,909	23	107
1100	0.0511	$1.01\times 10^{-58}$	0.0392	$7.05\times 10^{-59}$	99,900	21	112
1200	0.0479	$1.89\times 10^{-56}$	0.0363	$1.97\times 10^{-56}$	108,890	26	107
1300	0.0445	$1.02\times 10^{-52}$	0.0334	$1.06\times 10^{-52}$	117,881	30	114
1400	0.0445	$1.63\times 10^{-56}$	0.0331	$2.03\times 10^{-56}$	126,872	37	112
1500	0.0443	$4.23\times 10^{-60}$	0.0328	$4.45\times 10^{-60}$	135,868	43	118
