# Supplementary figures and images for: On the Empirical Agreement Between Compression and Program-Execution Approaches to Algorithmic Complexity: A Controlled Study Using BDM
Source: Entropy (Basel). 2026 May 27;28(6):601. doi: 10.3390/e28060601 (PMC13297901; doi:10.3390/e28060601)

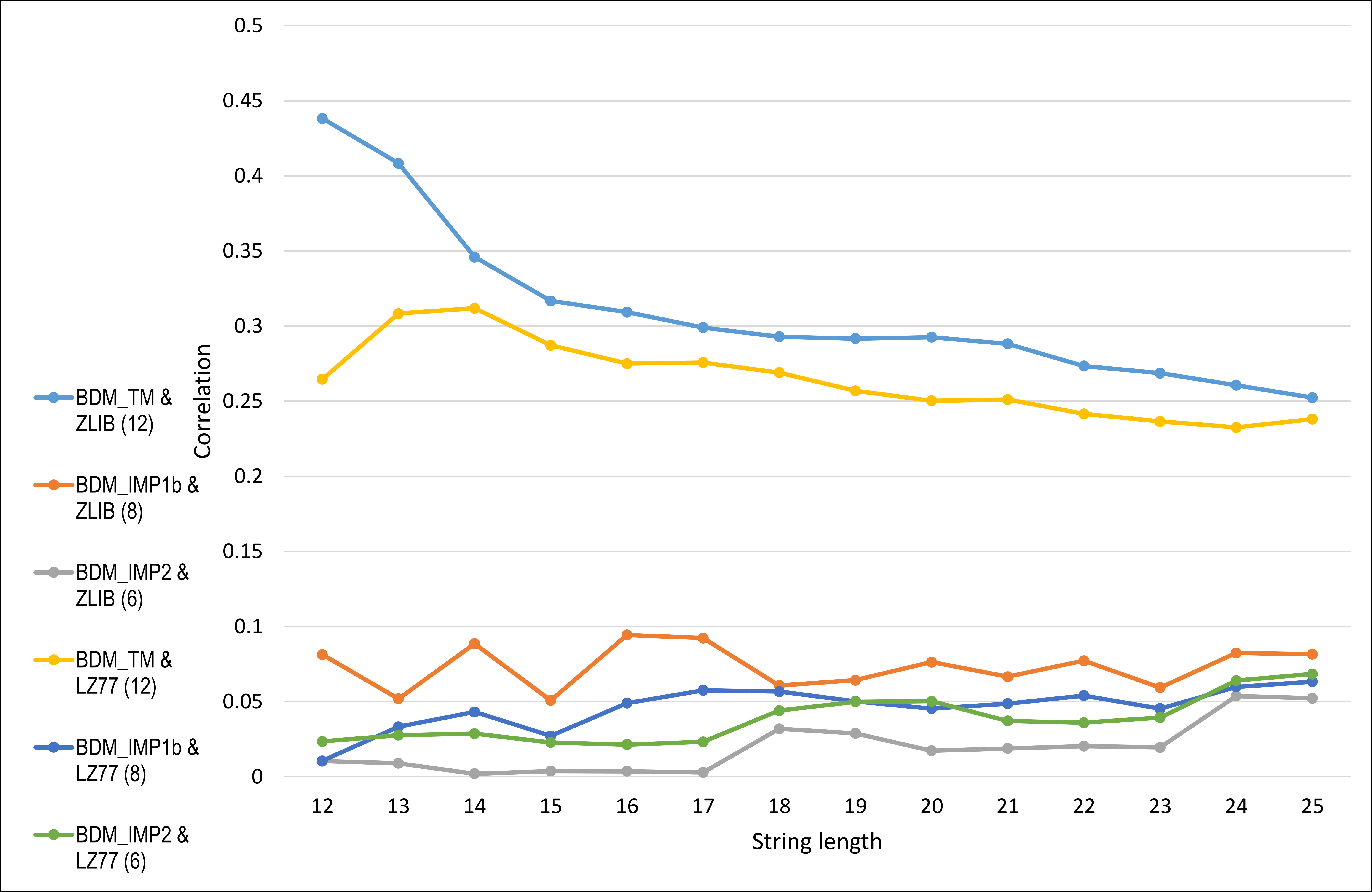

Supplement: Supplementary file 1 [file entropy-28-00601-s001.zip › spearman_exhaustive.png]

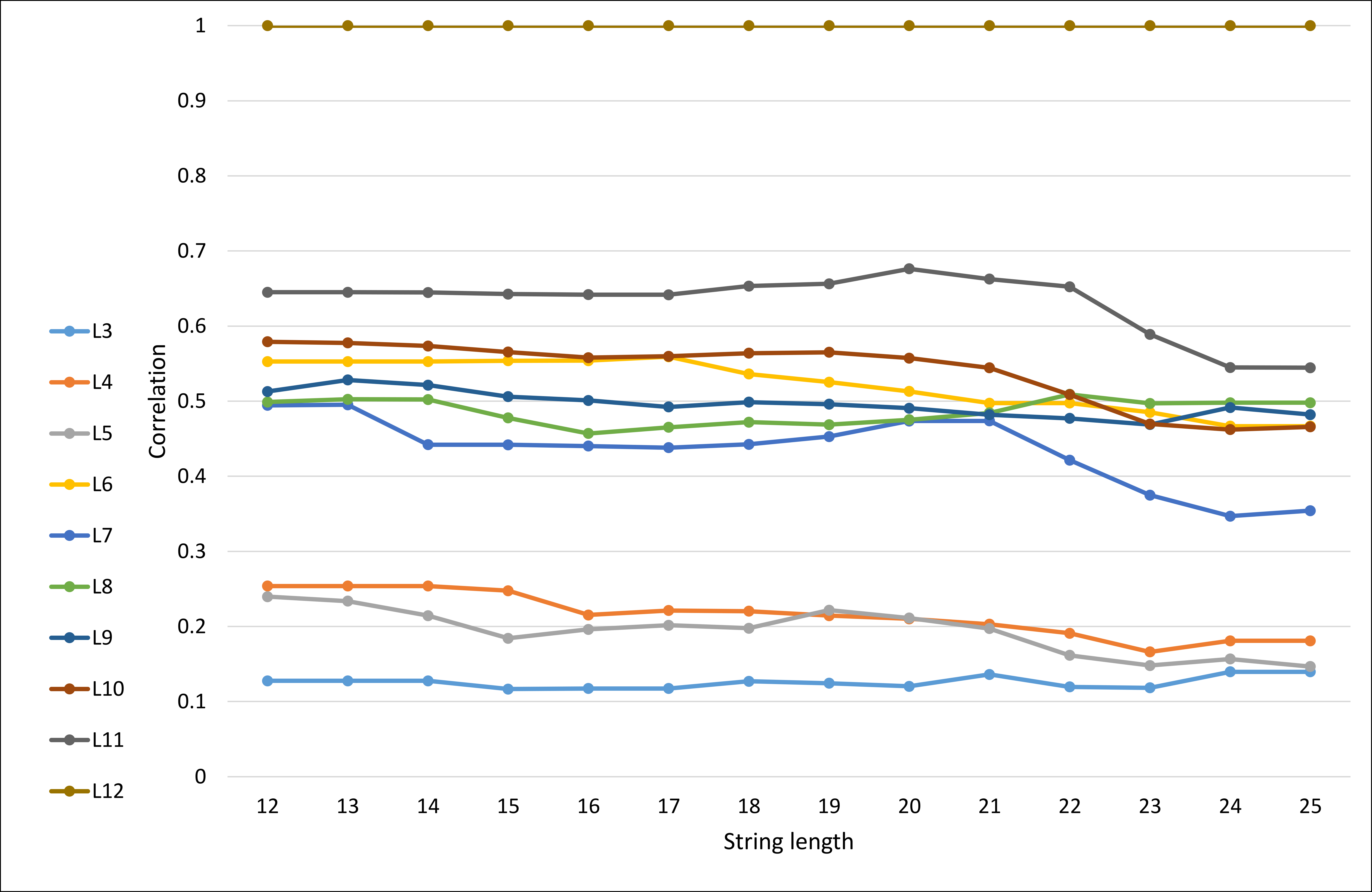

Supplement: Supplementary file 1 [file entropy-28-00601-s001.zip › stability_tm.png]
